# Bi-allelic variants in *FSD1L* cause a neurodevelopmental disorder overlapping with L1 syndrome

**DOI:** 10.1016/j.ajhg.2026.01.014

**Published:** 2026-02-19

**Authors:** Valentina Serpieri, Myriam Vezain-Mouchard, Alessia Orsi, Maryline Lecointre, Concetta Mazzotta, Florent Marguet, Anna Garbelli, Pascale Marcorelles, Ludovica Celli, Alice Goldenberg, Roberta De Mori, Nathalie Drouot, Francesco Petrizzelli, François Janin, Gaël Nicolas, Noor Smal, Claudia Condoluci, Carla Marini, Frederic Tran-Mau-Them, Valentin Ruault, Alessia Micalizzi, Silvia Bione, Tommaso Mazza, Anna Pichiecchio, Monia Ginevrino, Sarah Weckhuysen, Alice Bedois, Béatrice Desnous, Laurent Hermitte, Grace Rabie, Moien Kanaan, Bruno J. Gonzalez, Simone Sabbioneda, Annie Laquerrière, Pascale Saugier-Veber, Enza Maria Valente

**Affiliations:** 1 Department of Molecular Medicine, University of Pavia, 27100 Pavia, Italy; 2 Univ Rouen Normandie, INSERM U1245 and CHU Rouen, Department of Genetics and Reference Center for Developmental Disorders, 76000 Rouen, France; 3 Univ Rouen Normandie, INSERM U1245, 76000 Rouen, France; 4 Univ Rouen Normandie, INSERM U1245 and CHU Rouen, Department of Pathology, 76000 Rouen, France; 5 Institute of Molecular Genetics 'Luigi Luca Cavalli-Sforza', National Research Council (IGM-CNR), 27100 Pavia, Italy; 6 CHU Brest, Department of Pathology, 29200 Brest, France; 7 CH Le Havre, Department of Genetics, 76600 Le Havre, France; 8 Induced Pluripotent Stem Cells and Organoids Unit, IRCCS Santa Luicia Foundation, 00179 Rome, Italy; 9 Bioinformatics Laboratory, IRCSS Casa Sollievo Della Sofferenza, 71013 S. Giovanni Rotondo (FG), Italy; 10 Translational Epilepsy Genomics Group, VIB Center for Molecular Neurology, VIB, 2610 Antwerp, Belgium; 11 Developmental Disabilities and Rehabilitation, IRCCS San Raffaele Roma, 00166 Rome, Italy; 12 Child Neurology and Psychiatric Unit, G. Salesi Pediatric Hospital, Azienda Ospedaliera-Universitaria Delle Marche, 60126 Ancona, Italy; 13 Unité Fonctionnelle Innovation en Diagnostic Génomique des maladies rares, CHU Dijon Bourgogne, and INSERM UMR1231 GAD, 21079 Dijon, France; 14 Reference Center for Rare Diseases Developmental Anomaly and Malformative Syndromes, Genetics Department, Montpellier Hospital, 34000 Montpellier, France; 15 Laboratory of Medical Genetics, Translational Cytogenomics Research Unit, Bambino Gesù Children’s Hospital, IRCCS, 00146 Rome, Italy; 16 Medical Genetics Unit, San Pietro Fatebenefratelli Hospital, 00189 Rome, Italy; 17 Computational Biology and Bioinformatics Unit, Fondazione Policlinico Universitario Agostino Gemelli IRCCS, 00168 Rome, Italy; 18 Department of Brain and Behavioral Sciences, University of Pavia, 27100 Pavia, Italy; 19 Neuroradiology Department, IRCCS Mondino Foundation, 27100 Pavia, Italy; 20 Parkinson and Movement Disorders Unit, Study Center on Neurodegeneration (CESNE), Department of Neurosciences, University of Padua, 35121 Padua, Italy; 21 Translational Neurosciences, Faculty of Medicine and Health Science, University of Antwerp, 2610 Antwerp, Belgium; 22 Department of Neurology, University Hospital, 2650 Antwerp, Belgium; 23 Service de Génétique, Eurofins Biomnis, Lyon, France; 24 Department of Pediatric Neurology, Aix-Marseille University, Marseille, France; 25 Neuroradiology Department at Timone Hospital, Aix-Marseille University, Marseille, France; 26 Hereditary Research Laboratory, Bethlehem University, Bethlehem, Palestine; 27 Neurogenetics Research Center, IRCCS Mondino Foundation, 27100 Pavia, Italy

**Keywords:** *FSD1L*, L1 syndrome, *FSD1*, *L1CAM*, congenital hydrocephalus, corpus callosum, commissural axon navigation, microtubules, primary cilium, mitosis

## Abstract

Disruption of the complex processes underlying central nervous system development leads to a broad spectrum of brain malformations and neurodevelopmental disorders, often with a genetic cause. Here, we report bi-allelic pathogenic variants in fibronectin type III and SPRY domain-containing 1-like (*FSD1L*), encoding a protein of unknown function, in eleven individuals, including five fetuses from six unrelated families. The phenotype ranges from severe hydrocephalus, corpus callosum agenesis, and absent pyramid decussation to a neurodevelopmental syndrome characterized by severe intellectual disability, spastic tetraparesis, reduced vision, and epilepsy, associated with corpus callosum agenesis/hypoplasia, mild ventricular dilation, optic nerve hypoplasia, and white matter reduction. This phenotype closely resembles that observed in L1 syndrome, caused by pathogenic variants in *L1CAM*, encoding a neural adhesion molecule. The knockdown of *Fsd1l* in mouse embryos recapitulated the ventricular dilation observed in affected fetuses. Immunohistochemical studies in human control fetuses revealed that FSD1L localized to neurons with commissural fate and projection neurons during human development. Induced pluripotent stem cell (iPSC)-derived neural progenitor cells from affected individuals failed to differentiate into premature neurons and to properly form neurospheres while undergoing increased cell death. In neural progenitors, FSD1L localized with microtubules of the mitotic spindle during M phase and to the transition zone and along the axoneme of the primary cilium during interphase. In line with this, fibroblasts from affected individuals exhibited marked alterations of the mitotic spindle and reduced ciliogenesis and ciliary length compared to control cells. Our findings define FSD1L as a microtubule-associated protein implicated in neuronal differentiation, axon guidance, and fasciculation.

## Teaser

This study associates bi-allelic variants in *FSD1L* with a neurodevelopmental disorder closely resembling L1 syndrome, mainly characterized by hydrocephalus and corpus callosum defects. Functional experiments demonstrate that *FSD1L* encodes a microtubule-associated protein implicated in neuronal differentiation, cell division, cilia formation, axon guidance, and fasciculation.

## Introduction

The development of the human central nervous system (CNS) is an intricate and tightly regulated process involving sequential and overlapping stages of neural proliferation, migration, differentiation, and connectivity. Disruption at any of these stages can give rise to a wide spectrum of congenital brain malformations, which represent a major cause of neurodevelopmental defects. CNS malformations may variably affect cortical, midline, and hindbrain structures, reflecting the timing and nature of the developmental insult.^1^^,^^2^ Hydrocephalus often presents alongside complex brain malformations, either as a primary defect or as a secondary consequence of disrupted brain architecture.^3^

Over the past few decades, a growing number of genetic syndromes have been identified, associated with pathogenic variants in genes encoding for key players in brain development. Notably, defects in distinct classes of proteins may result in overlapping phenotypes due to convergent mechanisms. A clear example is the dysfunction of multiple pathways resulting in agenesis/dysgenesis of the corpus callosum, the main interhemispheric commissure mediating the transfer of information between the left and right hemispheres, which is crucial for bilateral integration of lateralized sensory, motor, and associative functions.^4^ The formation of the corpus callosum is orchestrated by a combination of molecular guidance cues and specialized glial structures that coordinate the successful navigation of axons across the midline.^5^^,^^6^^,^^7^ Disruption in these mechanisms—whether through altered localization of guidance molecules, defective glial scaffolding, or intrinsic axonal pathfinding errors—may result in the failure of axons to reach their appropriate destination. In line with this, callosal agenesis or dysgenesis is found in a spectrum of genetic conditions implicating diverse classes of proteins, such as cytoskeletal proteins (e.g., tubulins), neural adhesion molecules (e.g., L1CAM), proteins of the primary cilium (e.g., KIF7), and transcription factors (e.g., PAX6), all involved at different levels in axonal navigation.^8^

Here, using exome sequencing, we identified bi-allelic pathogenic variants in *FSD1L* (fibronectin type III [FnIII] and SPRY domain-containing 1-like; MIM: 609829) in eleven individuals from six unrelated families presenting a complex neurodevelopmental disorder and brain malformations resembling L1 syndrome (MIM: 307000 and 303350), a spectrum of conditions caused by pathogenic variants in *L1CAM* (MIM: 308840).^9^^,^^10^^,^^11^ We provide genetic and functional evidence that FSD1L is a microtubule-associated protein (MAP) implicated in mitotic spindle assembly and primary cilium formation, whose impairment hampers proper neuronal differentiation.

## Subjects, material, and methods

### Subjects

**Figure 1:**
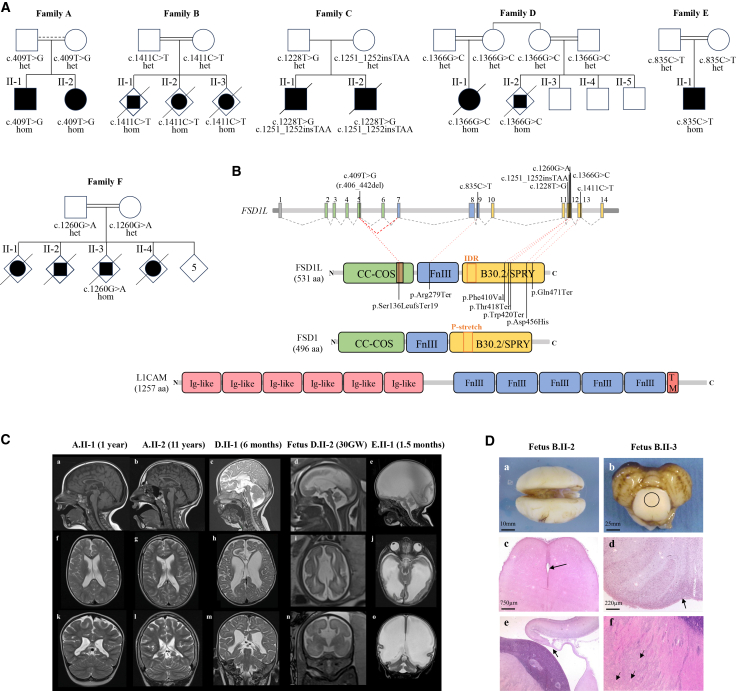
Bi-allelic pathogenic variants in *FSD1L* give rise to a neurodevelopmental disorder resembling L1 syndrome (A) Simplified pedigrees of the six families, A–F, with identified variants and familial segregation. Possible consanguinity is indicated with a dashed line. For family F, only the proband’s nuclear family is shown. (B) Schematic representation of *FSD1L* and its protein with location of the identified variants (annotated on GenBank: NM_001145313) and alignment with FSD1 and L1CAM proteins. FnIII, fibronectin type III domains; IDR, intrinsically disordered region; Ig-like, immunoglobulin-like domains; TM, transmembrane region. (C) Brain MRI of individuals A.II-1, A.II-2, D.II-1, and E.II-1 and fetus D.II-2, with sagittal T1-weighted (a and b), sagittal T2-weighted (c–e), axial T2-weighted (f–j), and coronal T2-weighted (k–o) sequences. Both A.II-1 and A.II-2 have a thin corpus callosum (a and b), while D.II-1 and D.II-2 show corpus callosum agenesis (c and d). In fetus D.II-2, parallel lateral ventricles (i), elevated 3^rd^ ventricle, and Probst bundles (n) are also evident. Individuals A.II-1, A.II-2, and D.II-1 demonstrate enlargement of the lateral ventricles (mainly in D.II-1) with irregular margins and “squared-off” trigones (f–h and k–m). They also show loss of periventricular, mainly peritrigonal, white matter (f–h and k–m), with a slightly increased T2 signal in A.II-1 and A.II-2 (g and h). Prominent sulci are also evident adjacent to the trigone of the lateral ventricles (f–h and k–m), and a slight cerebellar vermis hypoplasia is evident in individuals A.II-2 and D.II-1 and fetus D.II-2 (b–d), associated with mild brainstem hypoplasia in individuals D.II-1 and E.II-1 (c and e). E.II-1 also shows a massive supratentorial hydrocephalus with thinning of the bi-hemispheric cerebral cortex (j and o) and a corpus callosum, which is not visible (e); no flow artifact is seen in the Sylvian aqueduct (e), and there is a potential membranous web/adhesion at this level (j). (D) Main neuropathological characteristics of brains from fetuses B.II-2 and B.II-3. The macroscopic superior view of the brain showed the absence of corpus callosum (a) with macroscopically undiscernible aqueduct of Sylvius in the mesencephalon (b, black circle); the aqueduct of Sylvius could only be identified at the histological level and consisted of a severely narrowed lumen corresponding to true stenosis, with no forking and/or atresia (c, arrow). There was an absence of pyramid decussation at the bulbo-cervical junction (d, arrow). A section passing through the anterior diencephalon showed complete agenesis of the corpus callosum, without any Probst bundles (e, arrow), associated with severe dysplasia of the anterior arm of the internal capsule, made of several fascicles of varying size spreading into the deep gray nuclei (f, arrows).

This project includes eleven affected individuals from six unrelated families, recruited through GeneMatcher^12^ (families A–F, Figure 1A). Available clinical features and neuroimaging were collected from the referring clinicians. Written informed consent was obtained from all families participating in this study. The project was approved by the ethics committees of the University of Pavia (20180077857, date 12/09/2019) and the University Hospital of Rouen (biological collection number DC-2008-711).

### Neuropathological evaluation

A complete autopsy was performed in two fetuses from family B (B.II-2 and B.II-3) with informed written consent of the parents, in accordance with French law and following standardized protocols. Fetal biometric data were evaluated as reported.^13^ The brains were fixed in a 10% formalin-zinc buffer solution for at least 1 month. Brain growth and macroscopic assessment of brain maturation were evaluated according to the criteria of Guihard-Costa and Larroche and of Feess-Higgins and Larroche.^14^^,^^15^ Multiple 8-μm sections obtained from paraffin-embedded material (inclusion *in toto*) were stained using hematoxylin and eosin.

### Genetic analysis

Exome sequencing was performed on genomic DNA extracted from peripheral blood or amniotic cells, using either SureSelect Human All Exon kit v.6 (Agilent Technologies, Santa Clara, CA, USA) or Human Core Exome Panel (Twist Biosciences, South San Francisco, CA, USA). Average coverage ranged from 80× to 100×, with at least 98% of nucleotides covered by ≥10×. Bioinformatic analysis and variant annotation were carried out as previously reported.^16^^,^^17^ Variants were prioritized and filtered based on a read depth > 10×, a quality score ≥ 30, gnomAD allelic frequency < 0.1%, recessive inheritance, and, for missense variants, *in silico* predictions of pathogenicity (Table S1). The potential impact of identified missense variants on splicing was assessed by Human Splicing Finder (http://www.umd.be/HSF/),^18^ SpliceAI, and Pangolin (https://spliceailookup.broadinstitute.org/),^19^^,^^20^ while the impact of missense variants on protein dynamics and conformational transitions was evaluated by the Gaussian accelerated molecular dynamics simulation method (details in the supplemental material and methods).^21^

*FSD1L* variants were confirmed and segregated by conventional Sanger sequencing and have been deposited in LOVD database (https://www.lovd.nl/; accession numbers: IDs #00465958, #00465959, #00465960, #00465961, #0001059814, and #0001061021).

### RNA studies

Skin-derived primary fibroblasts were obtained from three individuals with FSD1L variants (A.II-1, A.II-2, and C.II-1) and three unrelated control subjects (HDF108, HDF109, and HDF204). Total RNA was extracted from fibroblasts (Euroclone Direct-zol RNA MiniPrep Plus, Pero, Italy) and from brain slices from the B.II-2 fetus and one control fetus (Maxwell CSC RNA FFPE kit, Promega, Charbonnière-les-bains, France) and retrotranscribed.

To evaluate the impact of the *FSD1L* c.409T>G variant on splicing, a fragment encompassing the variant was PCR amplified from cDNA, visualized on an agarose gel, and subsequently cloned and Sanger sequenced (details are provided in the supplemental material and methods).

To assess nonsense-mediated RNA decay on cDNA obtained from individuals A.II-1, A.II-2, and C.II-1, quantitative RT-PCR was performed as described,^22^ with *GAPDH* as the internal control. Due to the very limited amount of retrieved RNA from the fetal brain and the high level of degradation, only a semiquantitative PCR could be performed to amplify a fragment of cDNA from fetus B.II-2, with *NOTCH1* as the internal control. All conditions and primers are available upon request.

### Immunohistochemical analyses of FSD1L and L1CAM in the brain and eye of control and affected fetuses

The physiological localization of FSD1L in the brain and eye during fetal development was evaluated by immunohistochemistry in seven control fetuses (collection number DC-2015-2468, accession number AC-2015-2467) located at the Pr A. Laquerrière Pathology Laboratory, Rouen University Hospital (Table S2). Next, localization of FSD1L and L1CAM was assessed in two fetuses with *FSD1L* variants from family B (fetuses B.II-2 and B.II-3) and compared to two male fetuses interrupted at around 22 weeks of gestation (WG) carrying truncating pathogenic variants in *L1CAM* (details are provided in the supplemental material and methods). Primary and secondary antibodies used in this manuscript are reported in Table S3.

### Fsd1l-CRISPR-Cas9 *in utero* electroporation

Experiments were performed under the supervision of authorized investigators (authorization no. APAFIS#22136–2019092013438607 v.4 from the French Ministry of Health Research and Innovation). Briefly, the Fsd1l-CRISPR-Cas9 knockout (KO) plasmid consisted of a pool of three target-specific vectors, each encoding the Cas9 nuclease, and a guide RNA targeting 20 nt designed to KO *Fsd1l* expression. Unilateral intraventricular injections of a combination of Fsd1l-CRISPR-Cas9 KO plasmid and PCIG2-IRES-GFP plasmid (Fsd1l-CRISPR/GFP-electroporated group) or PCIG2-IRES-GFP plasmid alone (GFP-electroporated group) were performed on the brains of embryonic day (E)15 pregnant mouse embryos. Plasmid maps are shown in Figure S1. Three days after *in utero* electroporation (E18), the brains were collected for anatomical, immunohistochemical, and image analyses. Detailed methods for *in utero* electroporation and statistical analyses are reported in the supplemental material and methods.

### Cell models

Fibroblasts from three affected subjects (A.II-1, A.II-2, and C.II-1) and three healthy control subjects (HDF108, HDF109, and HDF204) were cultured in Dulbecco’s modified Eagle’s medium supplemented with inactivated 10% fetal bovine serum, 200 mM 1% L-glutamine, and 100× 1% penicillin/streptomycin (all from Euroclone, Pero, Italy), at 5% CO_2_ and 37°C.

Induced pluripotent stem cells (iPSCs) from two control subjects (HDF108 and HDF109) were obtained and characterized previously.^23^ To obtain iPSCs from affected individuals and the third control subject, fibroblasts were transduced using the CytoTune-iPS 2.0 Sendai Reprogramming Kit (Life Technologies, Carlsbad, CA, USA), according to the manufacturer’s protocol. Characterization of the obtained iPSC lines was performed as reported.^23^

Differentiation of iPSCs into neural progenitor cells (NPCs) and, subsequently, neuronal cells was carried out by adapting two available protocols, starting from 1.5 × 10^5^ iPSCs and 0.8 × 10^5^ NPCs, respectively (https://www.thermofisher.com/it/NPCs; https://www.thermofisher.com/it/neuronal differentiation).

To generate a knockin line endogenously expressing HA-tagged *FSD1L*, the CRISPR-Cas9 technique was employed to insert the HA sequence in frame at the 3′ end of one *FSD1L* allele in a control iPSC line (HDF109), using the Nucleofector 2b and Human Stem Cell Starter kit (Lonza, Basilea, Switzerland) (details are provided in the supplemental material and methods).

### Immunofluorescence

Immunofluorescence (IF) was performed as reported,^23^ after fixation with either cold methanol for 5 min (for fibroblasts) or paraformaldehyde (PFA) 4% for 15 min (for other cell types). Nuclei staining was performed with 300 nM DAPI and mounted with ProLong Gold antifade reagent (Thermo Fisher Scientific, Waltham, MA, USA). Primary and secondary antibodies used for IF are reported in Table S3.

### Analysis of neuronal differentiation

Differentiating NPCs were fixed with 4% PFA for 15 min on days 0, 2, 4, 8, and 12 for quantitative analysis of neuronal differentiation and cell death. IF experiments were performed using anti-βIII-tubulin and anti-HuC/D primary antibodies to mark the cytoskeleton and nuclei of premature neurons, respectively (Table S3). Two to four images were acquired at each time point using a confocal laser scanning microscope (Zeiss Confocal LSM-800). Images were processed with CellProfiler 4.1.3 (https://cellprofiler.org/),^24^ employing a custom pipeline to identify the proportion of cells positive for HuC/D over the total number of cells, using a nuclear mask. Data were plotted and analyzed using a two-way analysis of variance (ANOVA), with GraphPad Prism 6.0. Cell death was evaluated on day 4 by counting the proportion of condensed/fragmented nuclei over the total number of nuclei using Fiji/ImageJ.^25^ Three biological replicates were performed.

Neurosphere formation was assessed as described,^26^ by measuring the neurospheres’ diameter, as well as the distance that the farthest neuronal cell body traveled from the edge of the neurosphere, using Fiji/ImageJ.^25^ Experiments were performed in triplicate, and statistical analysis was carried out using the Student’s *t* test.

### Analysis of the mitotic spindle and nuclear anomalies

Analysis of the mitotic spindle was performed by adapting a published protocol,^27^ starting from 0.8 × 10^5^ fibroblasts seeded in 12-well plates with coverslips. Upon fixing cells in the M phase, mitotic spindles were visualized by IF using anti-α-tubulin and anti-histone-H2A primary antibodies (Table S3). For each cell line, the percentage of aberrant mitotic spindles was calculated out of 50 total spindles, and each experiment was performed in biological triplicate. The inter-pole distance was measured using Fiji/ImageJ.^25^ Nuclear anomalies were evaluated upon DAPI staining by counting the proportion of abnormal nuclei (e.g., hollow, faded, and multilobate nuclei) over the total number of nuclei in 10 images per coverslip, as reported.^28^ Statistical analysis was performed using a two-tailed Student’s *t* test.

### Analysis of primary cilia

Analysis of primary cilia in fibroblasts was performed upon starvation, as reported.^29^ Primary cilia were visualized by IF using anti-acetylated tubulin and anti-γ-tubulin antibodies to mark the ciliary axoneme and the centrosome, respectively (Table S3). The percentage of ciliated cells was calculated by dividing the number of cells showing a primary cilium by the total number of cells in each field, acquiring 15 images per coverslip. Ciliary length was measured from the centrosome to the tip of the cilium, measuring at least 100 cilia per coverslip. Each experiment was performed in triplicate. Statistical analysis was performed using the two-tailed Student’s *t* test.

## Results

### Exome sequencing identified bi-allelic variants in *FSD1L* in eleven affected individuals

As part of two parallel projects ongoing in Pavia and Rouen, aimed at unraveling genetic causes for neurodevelopmental disorders and congenital hydrocephalus, we independently identified candidate bi-allelic variants in *FSD1L* (GenBank: NM_001145313) in two siblings and in three fetuses from unrelated families A and B. Six additional affected individuals from families C, D, E, and F were identified through GeneMatcher.^12^ Variants were segregated from heterozygous healthy parents in all families (Figure 1A). Consanguinity was confirmed in families B, D, E, and F and suspected in family A.

Affected individuals from families A and D were homozygous for two distinct, apparently missense variants: c.409T>G (p.Leu137Val) (family A) and c.1366G>C (p.Asp456His) (family D). Despite being annotated as missense, c.409T>G was shown at the RNA level to impact splicing (r.406_442del), resulting in predicted frameshift and premature truncation: p.Ser136LeufsTer19 (see details later).

The two siblings from family C were compound heterozygous for the missense variant c.1228T>G (p.Phe410Val) and the nonsense variant c.1251_1252insTAA (p.Thr418Ter). Finally, the three affected fetuses from family B, the affected child from family E, and the affected fetus from family F were homozygous for three distinct nonsense variants: c.1411C>T (p.Gln471Ter) (family B), c.835C>T (p.Arg279Ter) (family E), and c.1260G>A (p.Trp420Ter) (family F) (Figure 1A).

No other relevant variants survived filtering in any of the affected individuals; in particular, no pathogenic variants in *L1CAM* or in genes related to tubulinopathies and ciliopathies were detected.

*FSD1L* is homolog of *FSD1* (MIM: 609828), and the proteins encoded by these two genes share a CC-COS (Coiled-coil C-terminal subgroup One Signature) domain, a FnIII domain, and a B30.2/SPRY domain. Different from FSD1, FSD1L lacks the p-stretch, a 20-aminoacid region highly subjected to phosphorylation, but contains two phosphorylable serine residues in positions 520 and 523.^30^ The FnIII domain is also shared with L1CAM (Figure 1B). No human phenotype associated with pathogenic variants in *FSD1L* or *FSD1* is currently known.

### *FSD1L* variants impact expression levels, protein length, or stability

*FSD1L* shows moderate intolerance to loss-of-function variation (loss-of-function observed/expected upper bound fraction [LOEUF] score = 0.84 in gnomAD), which is consistent with autosomal-recessive inheritance. All *FSD1L* variants are absent from gnomAD (v.4.1.0) except three, present at very low frequency and always in the heterozygous state: c.409T>G and c.1260G>A, detected in one and four European non-Finnish alleles, respectively, and c.835C>T, reported in eight alleles from individuals of multiple ancestries. The two missense variants c.1228T>G (p.Phe410Val) and c.1366G>C (p.Asp456His) were predicted as damaging or deleterious by most prediction software (Table S1). To further assess the impact of these variants on the protein’s structure and stability, we performed Gaussian accelerated molecular dynamics simulations. Analysis of the root-mean-square deviation (RMSD) profiles showed that the wild-type protein reached a stable conformation around ∼0.8 nm for the majority of the simulation time, whereas both mutant proteins exhibited higher fluctuations and only partial stabilization (Figure S2A). We next computed dynamical cross-correlation maps (DCCMs) to evaluate long-range interactions between all pairs of atoms. Excluding the highly flexible loop (residues 320–360), both mutant proteins showed altered covariance, especially in the FnIII domain, suggesting a long-range impact of the variants (Figure S2B). Finally, comparing the contact frequency observed during the simulations, both variants were found to alter the interaction network of neighboring residues (data not shown).

Variants c.409T>G, c.1251_1252insTAA, and c.1411C>T could be further assessed at the RNA level. To demonstrate the splicing defect predicted by bioinformatic tools (Figures S3A–S3C), a fragment encompassing variant c.409T>G was PCR amplified from cDNA extracted from fibroblasts. Samples from both affected individuals yielded a single band that was shorter and much less intense than the control band. Cloning and sequencing of the fragment confirmed that cDNA underwent aberrant splicing, with a loss of 37 nt of exon 5 (r.406_442del) (Figures S4A and S4B). This is predicted to result in a frameshift and introduction of a premature stop codon early in the protein: p.Ser136LeufsTer19. In line with this, quantitative RT-PCR showed a ∼95% reduced expression of *FSD1L*, indicating that the majority of this transcript underwent nonsense-mediated mRNA decay, while cDNA expression levels were comparable to control subjects in fibroblasts from affected individual C.II-1, heterozygous for the truncating variant c.1251_1252insTAA (Figure S4C). Upon PCR amplification of a short fragment of *FSD1L* cDNA from brain slices of fetus B.II-2, only the control band (corresponding to the housekeeping *NOTCH1*) could be amplified, suggesting that the nonsense variant c.1411C>T triggers complete mRNA decay (Figure S4D).

### The phenotypic spectrum of affected individuals resembles L1 syndrome

Six individuals were diagnosed postnatally, while in five fetuses, the pregnancy was terminated due to the poor prognosis of the detected fetal abnormalities.

Clinical, imaging, and anatomical features of individuals with *FSD1L* variants broadly resembled the spectrum of L1 syndrome, a neurodevelopmental X-linked disorder caused by pathogenic variants in *L1CAM*.^10^^,^^11^

In living subjects from families A, C, and D, the phenotype was characterized by psychomotor delay and severe intellectual disability with absent speech, spastic tetraparesis, reduced vision, severe swallowing and feeding difficulties, and epilepsy. Progressive microcephaly occurred in affected individuals from families C and D, while head circumference was at the 3^rd^ centile in the two siblings from family A. Systemic features variably included unspecific dysmorphic features, scoliosis, tetralogy of Fallot, sinus bradycardia, and genital abnormalities. Brain MRI revealed corpus callosum agenesis or hypoplasia, mild to severe ventricular dilation with irregular margins, reduction of periventricular white matter, optic nerve hypoplasia, and mild cerebellar and brainstem hypoplasia. In family D, a pregnancy was terminated at 34 WG upon detection of callosal agenesis and cerebellar hypoplasia on fetal MRI (Figure 1C).

In families B, E, and F, the phenotype was characterized by severe prenatal hydrocephalus and undetectable corpus callosum, leading to elective termination of multiple pregnancies or a neonatal phenotype characterized by macrocephaly, signs of intracranial hypertension, and encephalopathy with a lack of acquisition of developmental milestones (Figure 1C). In family B, an autopsy performed on the second and third fetuses (both females) disclosed severe hydrocephalus with stenosis of the aqueduct of Sylvius, corpus callosum agenesis without any Probst bundles, absence of corticospinal tracts, hypoplasia/agenesis of the internal capsule, camptodactyly, and bilateral clubfoot, while the eyes and spinal cord appeared histologically normal (Figure 1D).

Clinical, imaging, and neuropathological features of affected individuals are detailed in the supplemental notes.

### FSD1L localizes to several cerebral structures during human development

*FSD1L*, located on chromosome 9q31.2, gives rise to seven different transcripts, including a non-coding one. According to expression databases such as GTEx (https://www.gtexportal.org/home/) and MOCA (Mouse Organogenesis Cell Atlas), *FSD1L* is strongly expressed in the developing and mature brain, across several neuronal and glial populations (Figure S5).

To better describe the localization of FSD1L during normal human brain development and compare it with L1CAM, immunohistochemical studies were undertaken in seven control fetuses in whom *FSD1L* pathogenic variants were excluded (Table S2).

**Figure 2:**
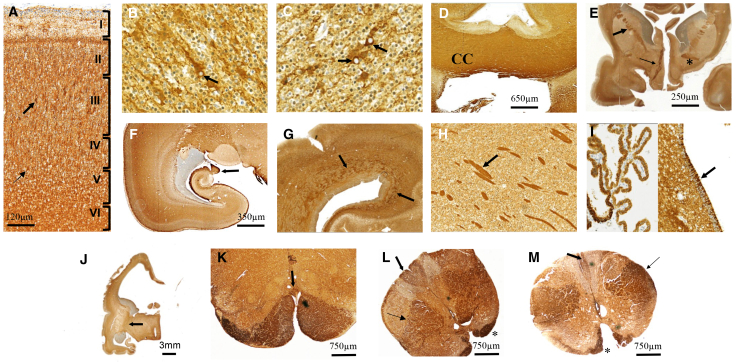
FSD1L immunoreactivity is broadly detected in the developing brain Immunohistochemistry of sections from control fetal brain using anti-FSD1L antibody. (A–C) FSD1L immunoreactivity was observed in the perikarya, the apical dendrites, and the neuritic network from neurons of layer III of the cortical plate (A and B, arrows), as well as in those of pyramidal neurons of layer V (C, arrows). (D–F) A strong immunoreactivity was also observed in the corpus callosum (D); in the anterior arm of the internal capsule (E, thick arrow), the anterior commissure (E, asterisk), and the fornical commissure (E, thin arrow); and at the level of the hippocampal uncus (F, thick arrow). (G and H) The periventricular fiber network was also immunoreactive (G, arrows), as well as the putamino-caudate fibers (H, arrow). (I) Notably, epithelial cells of the choroid plexuses and ependymal cells bordering the lateral ventricles were also immunoreactive. (J and K) Conversely, the posterior arm of the internal capsule was negative (J, arrow), whereas the pyramids and their decussation at the bulbo-cervical junction were strongly immunolabeled (K, arrow). (L and M) In the spinal cord, the anterior pyramidal tract was strongly immunolabeled (asterisk) and so were, to a lesser degree, the lateral pyramidal tracts (thin arrows) and the gracile fascicle (thick arrow).

From 12 to 22 WG, similar immunoreactivities were observed among all control fetuses. In the cortical plate, the perikarya of neurons undergoing differentiation were strongly immunoreactive, along with their apical dendrites and axons. In the fetus aged 22 WG, these neurons were located in layers III and V, corresponding to neurons with commissural fate and projection neurons (Figures 2A–2C). The supratentorial commissures, i.e., the corpus callosum, the anterior commissure, and the fornix, were also strongly immunolabeled (Figures 2D–2F). An intense immunoreactivity was noted within the periventricular network, which contains thalamo-cortical and cortico-subcortical afferents and afferents from the basal forebrain (Figure 2G). The anterior arm of the internal capsule, containing the anterior thalamic peduncle, the fronto-pontine fascicle, and the corticostriatal projections, as well as afferences and efferences of the deep gray nuclei, i.e., putamino-caudate, pallidal, and striato-nigral fibers, was also immunoreactive (Figures 2E–2H). Intense immunoreactivity was also detected in the ependymal lining of all ventricles and choroid plexuses (Figure 2I). Conversely, the posterior arm of the internal capsule, which is made up of axons of the pyramidal tracts, remained negative whatever the term (Figure 2J). The pyramids and their decussation at the bulbo-cervical junction were strongly immunoreactive (Figure 2K). In the spinal cord, FSD1L immunoreactivities were detected on the gracile fascicle and the anterior and lateral pyramidal tracts, but the neurons of the anterior and posterior horns were completely negative (Figures 2L and 2M). Except for the pyramidal tracts and the gracile fasciculus, the other tracts were negative. FSD1L immunoreactivity was also assessed in the optic nerve and retina. In control fetuses, almost all axons of the optic nerve were immunoreactive, while all layers of the sensorial retina were negative, even the ganglion cells, which send their axons to the optic chiasm via the optic nerve. In the eye, these axons form a layer located under the internal limitans membrane, which was strongly immunoreactive (Figures S6A and S6B).

**Figure 3:**
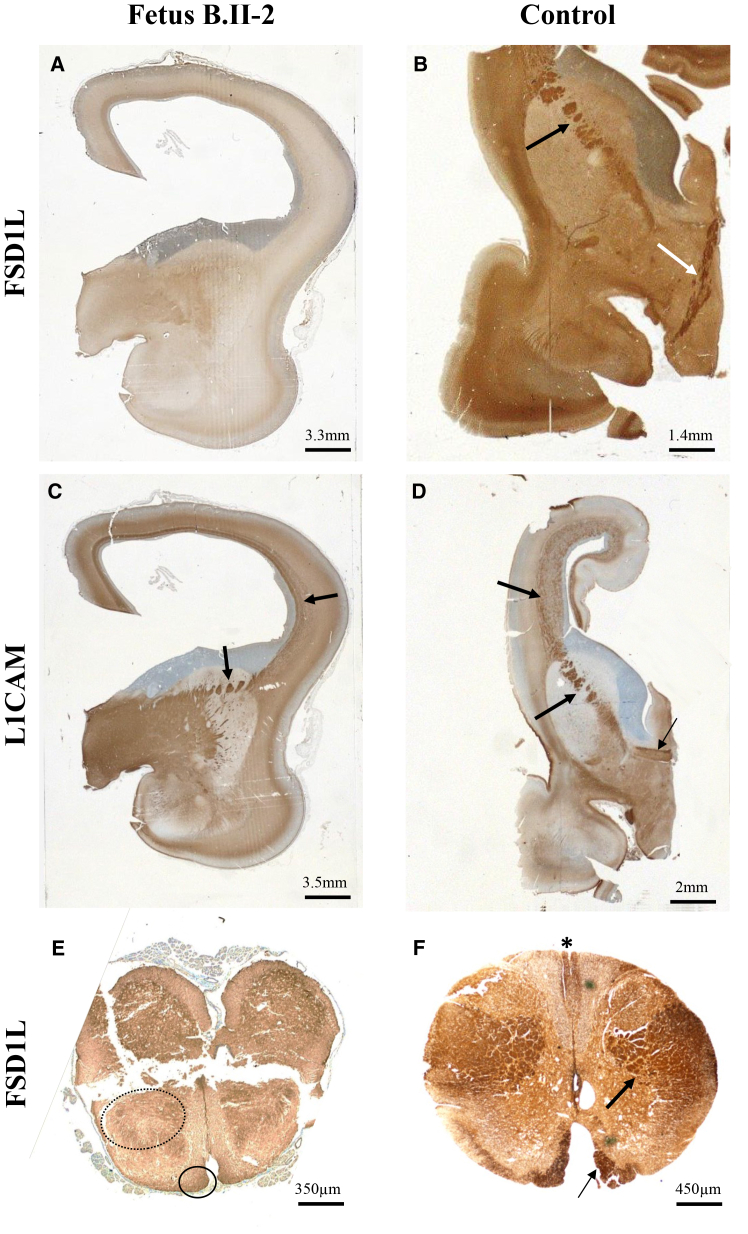
Absence of FSD1L immunoreactivity in the brain of affected fetuses (A and B) Absence of immunoreactivity in the anterior diencephalon of fetus B.II-2 (A) by comparison with an age-matched control brain in which the anterior arm of the internal capsule (B, black arrow) and the fornical commissure (B, white arrow) are strongly positive. (C and D) Conversely, in the affected fetus, the periventricular network and the anterior arm of the internal capsule are strongly immunolabeled by L1CAM antibody (C, arrows), similarly to the control (D, thick arrows), in which the anterior commissure is also immunoreactive (D, thin arrow). (E and F) The spinal cord of the affected fetus displays no FSD1L immunoreactivity (E, dotted and full circles), while in the control fetus, the anterior (F, thin arrow) and lateral (F, thick arrow) pyramidal tracts and the gracile fascicle (F, asterisk) are immunoreactive.

Comparative immunohistochemistry performed with anti-L1CAM antibodies revealed overlapping immunoreactivity patterns (Figures 3B–3D, 3F, S6A, S6B, S6G, and S6H).^11^

Unlike control fetuses, FSD1L immunoreactivity was absent in the brain, spinal cord, and eyes of the two fetuses with *FSDS1L* variants, where, instead, L1CAM was normally localized (Figures 3A–3C, 3E, S6C, S6D, S6I, and S6J). Conversely, FSD1L was normally detected in the brain, spinal cord, and eyes of two fetuses carrying *L1CAM* variants, while L1CAM immunoreactivity was absent (Figures S6E, S6F, S6K, and S6L; data not shown).

### *In utero* electroporation of Fsd1l-CRISPR/GFP plasmids reproduces ventricular enlargement observed in affected fetuses

We next knocked out *Fsd1l* in the brain of mouse embryos (E15) through *in utero* electroporation of a combination of Fsd1l-CRISPR-Cas9 KO and GFP-expressing plasmids. Since not all cells were electroporated, the functional result was a knockdown of *Fsd1l* in the electroporated area. At E18, several GFP-positive cells were detected in the intermediate zone (IZ), the cortical plate, and the developing striatum of the electroporated side of the brain, whereas the contralateral side was devoid of GFP-positive cells. At higher magnification, no colocalization was observed between GFP- and Fsd1l-positive cells, indicating that GFP-positive cells did not express *Fsd1l*. Surface plots of the GFP and Fsd1l intensity profiles revealed a strong reduction of Fsd1l fluorescence intensity in the IZ and striatum, which overlapped with the GFP-intensity map (Figure S7).

**Figure 4:**
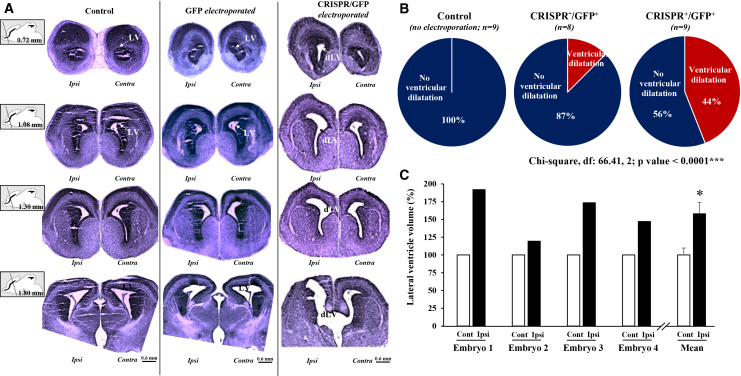
*Fsd1l* repression induces ventriculomegaly in E18 mouse embryos (A) Drawn sagittal planes of E18 mouse embryos where the black line illustrates the antero-posterior positioning of the histological slices and the number indicates the distance of the slice from the rostral position, as defined in the atlas of the developing mouse brain. Anteroposterior Cresyl violet-stained sections allow the visualization of the lateral ventricle (LV) morphology in control, GFP-electroporated, and Fsd1l-CRISPR/GFP-electroporated embryos. In the ipsilateral side of the Fsd1l-CRISPR/GFP-electroporated embryo, the lateral ventricle is dilated (dLV). (B) Frequency of ventriculomegaly occurring in the control (left), GFP-electroporated (middle), and Fsd1l-CRISPR/GFP-electroporated (right) groups. Electroporation of the Fsd1l-CRISPR/GFP plasmid mix at E15 resulted in a significant increase in the proportion of embryos presenting a dLV at E18. A statistical comparison of the three distributions was done using the chi-squared test. ^∗∗∗^*p* < 0.0001. (C) Graph comparing the volumes of ipsi- (dilated) and contralateral LVs in the four E18 embryos with ventriculomegaly. Statistical analysis was done on the mean values ± SEM with an unpaired *t* test. ^∗^*p* = 0.02, *n* = 4.

When compared to untransfected control mouse embryos, no significant alterations were found in the group of embryos electroporated with GFP-only plasmids. In particular, the morphology of lateral ventricles was similar in the ipsi- and contralateral sides of control and GFP-electroporated embryos, with only 13% showing asymmetric ventricles. In contrast, in the Fsd1l-CRISPR/GFP-electroporated group, 44% of embryos presented asymmetric lateral ventricle dilation (Figures 4A and 4B). Quantification of the lateral ventricular dilation in the Fsd1l-CRISPR/GFP-electroporated group indicated that the ventricular volume of the ipsilateral side was significantly larger than that of the contralateral side, supporting that Fsd1l repression is associated with ventriculomegaly (Figure 4C).

### FSD1L is required for proper neuronal differentiation

**Figure 5:**
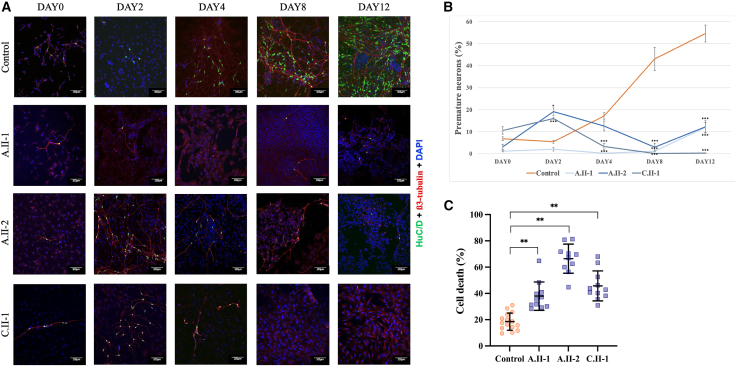
Neuronal precursors from affected individuals show impaired differentiation into premature neurons and increased cell death (A) Representative confocal images of NPCs from one control subject and individuals A.II-1, A.II-2, and C.II-1 along a 12-day differentiation protocol toward premature neurons. Cells are counterstained with antibodies against β3-tubulin (red) and HuC/D (green). Nuclei are stained with DAPI (blue); scale bar: 100 μM. (B) Graph showing the percentage of premature neurons (HuC/D positive) from control and affected individuals along the differentiation protocol (mean ± SE). (C) Graph showing the percentage of cell death (measured by counting condensed/fragmented nuclei) on day 4 of the differentiation protocol. ^∗^*p* < 0.05, ^∗∗^*p* < 0.01, and ^∗∗∗^*p* < 0.005.

To explore the functional impact of *FSD1L* variants at the neuronal level, iPSCs from three affected individuals (A.II-1, A.II-2, and C.II-1) and three healthy control subjects were differentiated into NPCs and then premature neurons over a 12-day time course. Control cells consistently showed a progressive increase in the proportion of premature neuronal cells over time. Conversely, a markedly impaired neuronal differentiation was evident in all three affected individuals’ lines, showing either a nearly complete absence of premature neurons throughout the time course or an initial attempt to differentiate followed by a loss of HuC/D-positive cells at later time points (Figures 5A and 5B). This failure in differentiation was paralleled by a significantly higher proportion of cell death, as shown by a 2- to 4-fold increase in the number of condensed/fragmented nuclei in affected individuals compared to control subjects on day 4 (Figure 5C).

**Figure 6:**
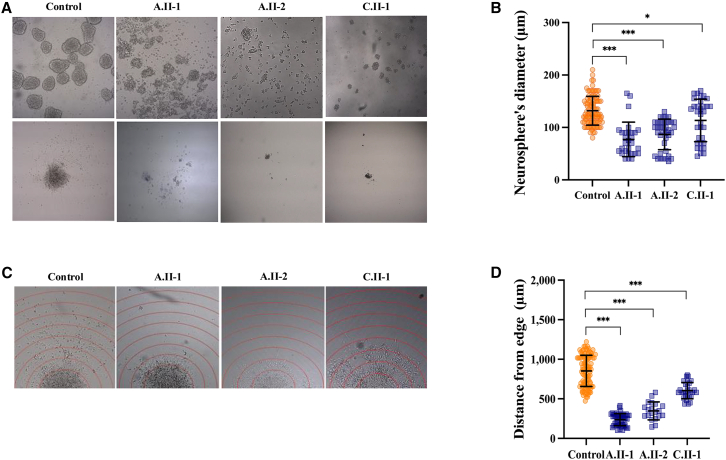
Neuronal precursors from affected individuals fail to form proper neurospheres (A) Representative images of neurospheres formed by differentiating NPCs of affected individuals vs. control subjects, showing marked disorganization in the non-adherence phase (top) and reduced adherence ability to a coated surface (bottom). (B) Quantitative evaluation of neurospheres' diameter in adherence phase, acquired at 4× magnification. (C and D) Representative images (C) and graph (D) showing measurement of the distance that the farthest neuronal cells traveled from the edge of the neurospheres. ^∗^*p* < 0.05, ^∗∗^*p* < 0.01, and ^∗∗∗^*p* < 0.005.

To confirm this, we tested the ability of NPCs to form neurospheres. Neurospheres from affected individuals appeared markedly disorganized and significantly smaller in size compared to control subjects (Figures 6A and 6B) and showed poor adhesion capacity to the surface. Moreover, we observed a drastic reduction in the distance traveled by the few neuronal cells exiting the neurospheres (Figures 6C and 6D).

### FSD1L associates with microtubules and is implicated in key microtubule structures

The canonical transcript of *FSD1L* (GenBank: NM_001145313) encodes for a TDARK protein of 531 amino acids, whose function is completely unknown. *FSD1*, the closest homolog of *FSD1L*, encodes for a protein that was found to localize to the centrosome and microtubule asters emerging from it, as well as to the basal body of the primary cilium, and to regulate axoneme assembly and ciliogenesis.^30^^,^^31^

**Figure 7:**
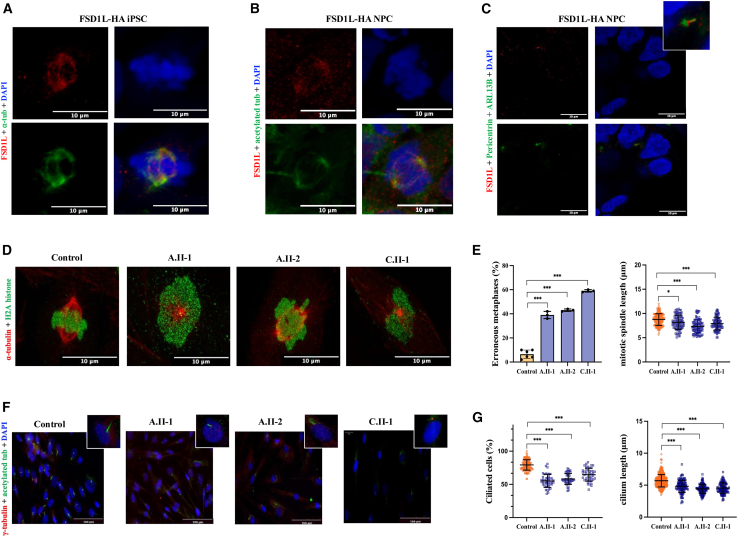
FSD1L associates with microtubules and is involved in the formation of the mitotic spindle and the assembly of the primary cilium (A) In iPSCs, endogenous FSD1L-HA shows association with microtubules of the mitotic spindle during M phase; scale bar: 10 μM. (B and C) In NPCs, FSD1L-HA associates with microtubules of the mitotic spindle during M phase (B) and is found at the transition zone and along the axoneme of the primary cilium during interphase (C); scale bar: 10 μM. (D) Representative images of defective mitotic spindles in fibroblasts of individuals A.II-1, A.II-2, and C.II-1 compared to control subjects; scale bar: 10 μM. (E) Graphs showing the mean ± standard deviation of the percentage of abnormal metaphases (left) and measurement of inter-polar distance (right). (F) Representative images of primary cilia in fibroblasts from individuals A.II-1, A.II-2, and C.II-1 compared to control subjects; scale bar: 100 μM. (G) Graphs showing the percentage of ciliated cells (left) and measurement of primary cilium average length (right) in fibroblasts from affected individuals vs. control subjects. ^∗^*p* < 0.05, ^∗∗^*p* < 0.01, and ^∗∗∗^*p* < 0.005.

To accurately evaluate the subcellular localization of FSD1L, we generated a control iPSC line in which one endogenous *FSD1L* allele was HA tagged at the C terminus and carried out IF experiments along different stages of the cell cycle using anti-HA antibody. While FSD1L-HA showed a diffuse punctate cytoplasmic staining in interphase in iPSCs, we observed a clear association with microtubules of the mitotic spindle (Figure 7A), which was more evident during prophase and metaphase and then disappeared during late anaphase. Upon differentiation of iPSCs into NPCs, in addition to the previously observed localization, FSD1L-HA also localized to the primary cilium during interphase, both at the transition zone and along the axoneme (Figures 7B and 7C).

Based on these observations, we employed fibroblasts from affected individuals to test whether FSD1L impairment could have a deleterious impact on the formation of the mitotic spindle and/or on the assembly of primary cilia. By comparison with control cells and upon synchronization of the cell cycle, fibroblasts from affected individuals displayed a significantly increased number of abnormal metaphases, which featured chromosomal misalignment, multipolar/monopolar spindles, lagging chromosomes, and impaired chromosomal compaction. Mitotic spindle length was also significantly reduced (Figures 7D and 7E). As expected, spindle defects were frequently associated with abnormal nuclear morphology, with a statistically significant increase of hollow, faded, and multilobed nuclei (Figure S8). Primary ciliogenesis upon 24-h starvation was also impaired in fibroblasts from affected individuals, which showed a significant reduction in the percentage of ciliated cells and the average ciliary length, compared to control subjects (Figures 7F and 7G).

## Discussion

We describe a neurodevelopmental syndrome associated with bi-allelic truncating or missense variants in *FSD1L* and provide functional insights into the role of this previously uncharacterized gene. The consistent phenotype observed in six unrelated families—ranging from prenatally detectable severe brain malformations to a postnatal syndrome presenting with severe intellectual disability, spastic tetraparesis, epilepsy, and reduced vision—and the demonstration that *Fsd1l* depletion in mouse embryos recapitulated the ventricular dilation observed in affected fetuses provide robust evidence for the involvement of *FSD1L* in this neurodevelopmental disorder.

The spectrum of brain malformations encompasses hydrocephalus, agenesis or hypoplasia of the corpus callosum, optic nerve and cerebellar hypoplasia, absent decussation of the pyramids, and reduced white matter. These defects mainly affect the areas in which *FSD1L* is mostly expressed during fetal life, in particular layers III and V of the cortical plate (corresponding to neurons with commissural fate and projection neurons), axons forming the supratentorial commissures, afferences and efferences of the deep gray nuclei, the ependymal lining of ventricles, the anterior arm of the internal capsule, the pyramidal tracts, and the optic nerve. Altogether, the prominent localization of FSD1L in axonal tracts that form commissures (such as the anterior commissure, corpus callosum, and fornical commissure) and in the decussation of the corticospinal tract at the bulbo-medullary junction and within the spinal cord suggests that FSD1L contributes to commissure formation and could be involved in axonal guidance during midline crossing.

The *FSD1L*-associated clinical, imaging, and neuropathological phenotypes, as well as its localization pattern in the developing brain, closely mirror those of *L1CAM*, which encodes a member of the immunoglobulin superfamily of cell adhesion molecules. Pathogenic variants in *L1CAM* give rise to L1 syndrome, which encompasses a spectrum of neurodevelopmental disorders, including hydrocephalus of variable severity, corpus callosum agenesis, cerebellar hypoplasia, agenesis of the pyramidal tracts, and, in living subjects, spasticity, severe intellectual disability with absent speech, and adducted thumbs.^9^^,^^10^^,^^11^ In both syndromes, the occurrence and severity of ventricular enlargement show marked variability among affected individuals, as also observed in mouse models of L1 syndrome according to their genetic background, suggesting the existence of genetic modifiers.^32^

L1CAM is localized on most developing axons and has been implicated in neuronal migration, neurite growth, and axonal fasciculation. It features an extracellular region with six Ig-like and five FnIII domains, a transmembrane region, and a cytoplasmic region.^33^ The FnIII domains, which are also found in FSD1L and FSD1, play key roles in cell adhesion and migration, allowing the establishment of protein-protein interactions. For instance, L1CAM can bind FGFR on the axon growth cone through the FnIII domains, triggering a downstream signaling cascade required for neurite outgrowth.^34^

Recent studies have also provided a direct link between neural cell adhesion molecules, microtubules, and MAPs—a family of proteins that regulate microtubule assembly, stability, and interaction with other cellular components—in order to convert extracellular signals into structural modifications that result in axonal growth.^35^ L1CAM was found to directly bind MAP2c, a MAP mainly detected in developing neurons, and to enhance its accumulation to promote neurite outgrowth.^36^ Furthermore, L1CAM can be endocytosed in axonal growth cones, localizing to vesicles along the microtubules before being reinserted into the plasma membrane, contributing to axonal growth cone mobility.^37^

Besides the FnIII domain, FSD1L and FSD1 also share two domains implicated in microtubule binding, the CC-COS domain and the B30.2/SPRY domain. While B30.2/SPRY is a versatile protein domain acting as an adaptor or scaffold to facilitate protein-protein interactions, the CC-COS domain is a specific structural motif crucial for microtubule binding. These domains are also found in MID1 and MID2, two MAPs that are mutated in Opitz syndrome, a neurodevelopmental disorder characterized by intellectual disability and midline anomalies, with corpus callosum agenesis and cerebellar vermian hypoplasia.^38^^,^^39^^,^^40^ Microtubules are filaments formed of dimers of α- and β-tubulin polymerized in a head-to-tail fashion, which play essential roles in diverse cellular functions such as intracellular transport; control of cell morphology, polarity, and migration; signal transduction; and cell division.^41^^,^^42^ The major microtubule-organizing center is the centrosome, formed by two barrel-like microtubule structures called centrioles. In dividing cells, centrosomes form the poles anchoring the mitotic spindle, essential for accurate chromosomal segregation, while in non-dividing cells, a modified centriole called the basal body acts as a template to build the primary cilium, whose main structure is also made of microtubules.^43^ The interaction of MAPs with microtubules is a dynamic process, with binding affinity varying across different stages of the cell cycle and usually regulated by specific modifications of MAPs, such as phosphorylation.^44^ Previous evidence already demonstrated that the FSD1L homolog FSD1 can dynamically bind to microtubules and that this binding is regulated by phosphorylation of specific residues. During interphase, endogenous FSD1 showed a pericentrosomal localization, while it dissociated from centrosomes at prophase, to reappear at the spindle poles in late anaphase and then localize to the midbody region in telophase. Different from the endogenous protein, overexpressed GFP-FSD1 did not localize to the centrosome but associated directly with microtubules, particularly on the microtubule asters stemming from the centrosome. In particular, GFP-FSD1 dissociated from microtubules during mitosis, assuming a diffuse cytoplasmic pattern, to reassociate with the midbody region at telophase. Both the CC-COS and B30.2/SPRY domains of FSD1 were found to contribute to microtubule binding.^30^ A subsequent study showed that FSD1 plays an essential role in anchoring microtubule asters to the basal body, promoting the assembly of the transition zone at the base of the primary cilium. In line with this, *FSD1* knockdown resulted in defective ciliogenesis and caused cilia-related phenotypes in zebrafish embryos.^31^

Based on these findings, we sought to explore whether FSD1L could also associate with microtubules and/or centrosomes. To avoid possible mislocalization related to protein overexpression, we engineered a control iPSC line by inserting an HA tag at the 3′ end of one copy of *FSD1L* and then visualized the endogenous protein with an anti-HA antibody. Different from FSD1, we observed colocalization with microtubules of the mitotic spindle during mitosis, both in iPSCs and upon differentiation into neural progenitors, which disappeared in late anaphase. In NPCs, FSD1L is also localized at the transition zone and along the axoneme of the primary cilium during interphase. Coherently with these cellular localizations, fibroblasts of affected individuals showed marked abnormalities of the mitotic spindle and impaired ciliation, implicating FSD1L in the regulation of these two essential cellular processes.

The assembly of the mitotic spindle is a highly sophisticated process requiring a complex regulation of microtubule dynamics, in which several MAPs have been involved.^45^ Mitotic defects are known to activate a control mechanism termed the spindle assembly checkpoint, triggering apoptosis.^46^ A group of disorders featuring mitotic spindle defects is primary microcephaly, mainly caused by pathogenic variants in genes encoding for centrosomal proteins. However, these conditions usually present a marked depletion of the pool of neural progenitors, resulting in fewer cells in the cerebral cortex, and are thus characterized by a severe reduction of brain size already present at birth or manifesting prenatally.^47^ Conversely, we did not observe a depletion of NPCs derived from cells from affected individuals, but neural precursors showed impaired ability to differentiate and form neurospheres, with persistence of immature cells lacking proper neuronal projections. Consistent with this observation, in living affected individuals, head circumference progressively decreased after birth, instead suggesting progressive neuronal loss. Of note, depletion of either MID1 or MID2 was also found to cause mitotic spindle defects, with nuclear abnormalities and increased cell death.^48^

Besides mitotic defects, we also observed impairment in the assembly of the primary cilium, resembling that observed in *FSD1*-KO cells.^31^ Primary cilia play multiple roles during brain development, such as the transduction of key signaling pathways, such as Shh and canonical Wnt.^49^^,^^50^ In contrast to primary ciliopathies, where functional defects of the primary cilium are the direct cause of specific multiorgan manifestations,^51^ ciliary anomalies can also result from mutations in proteins whose main function resides outside the ciliary compartment. In these “secondary ciliopathies,” the disease phenotype is primarily driven by extraciliary dysfunction, and ciliary defects represent collateral manifestations rather than the primary driver of the disease.^52^^,^^53^ While *FSD1L*-associated neurodevelopmental syndrome is likely to represent a secondary ciliopathy, it is worth noting that affected individuals with Joubert syndrome, the archetypal neurodevelopmental primary ciliopathy, can manifest brain malformations partly overlapping with those observed in individuals carrying *FSD1L* variants, such as a lack of pyramid decussation, hydrocephalus, and corpus callosum defects.^54^^,^^55^

This study has some limitations. First, the small number of affected individuals carrying missense variants likely restricted the ability to delineate the full phenotypic spectrum associated with *FSD1L* variants. Secondly, the mechanism underlying the progressive microcephaly observed in some children still remains to be explained. Finally, the unavailability of cells or tissue from individuals carrying bi-allelic truncating variants hindered the assessment of the cellular phenotype associated with the complete loss of function of *FSD1L*.

In conclusion, we describe a neurodevelopmental syndrome overlapping with L1 syndrome resulting from bi-allelic pathogenic variants in *FSD1L*, a gene highly expressed in the brain that encodes a protein associated with microtubule structures. Further studies are needed to reveal the pathogenetic mechanisms linking FSD1L disruption to the observed defects of neuronal differentiation and axonal navigation.

## Data and code availability

The accession numbers for *FSD1L* variants reported in this paper is deposited in LOVD Database.

## Acknowledgments

We are grateful to the affected individuals and families who contributed to the study. We also warmly thank Dr. Valérie Layet, who recruited family B several years ago, and Dr. Marta Romani, who contributed to the initial exome sequencing studies in family A. This work is dedicated to the memory of Prof. Giorgio Albertini, who first ascertained family A and got the whole project started. He prematurely passed away in 2017. This study was supported by Telethon and the Cariplo Foundation (grant GJC21046 to E.M.V.); by #NEXTGENERATIONEU (NGEU), funded by the Ministry of University and Research (MUR), National Recovery and Resilience Plan (NRRP), project MNESYS (PE0000006) – A Multiscale integrated approach to the study of the nervous system in health and disease (DN. 1553 11.10.2022); and by the European Union and Région Normandie in the context of Recherche Innovation Normandie (RIN 2018). Europe gets involved in Normandie with the European Regional Development Fund (ERDF). S.W. received funding from FWO (1861424N), and N.S. received funding from University of Antwerp-BOF (FFB180186). This work was generated within the European Reference Network for Developmental Abnormalities and Intellectual Disability.

## Author contributions

Conception and design of the study, V.S., M.V.-M., B.J.G., A.L., P.S.-V., and E.M.V.; execution of experiments and acquisition and analysis of data, VS, M.V.-M., A.O., M.L., C. Mazzotta, F.M., A. Garbelli, L.C., R.D.M., N.D., F.P., F.J., G.N., N.S., A.M., S.B., T.M., M.G., S.W., B.J.G., S.S., A.L., P.S.-V., and E.M.V.; recruitment, clinical, neuroimaging, and neuropathological characterization, F.M., P.M., A. Goldenberg, C.C., C. Marini, F.T.-M.T., V.R., A.P., A.L., A.B., B.D., L.H., G.R., and M.K.; manuscript writing, V.S., M.V.-M., G.N., B.J.G., A.L., P.S.-V., and E.M.V.; revision of the manuscript for intellectual content, all co-authors; project supervision, P.S.-V. and E.M.V.

## Declaration of interests

The authors declare no competing interests.

## Web resources

GenBank, https://www.ncbi.nlm.nih.gov/genbank/
OMIM, https://www.omim.org

## Supplemental information

Document S1. Figures S1–S8, Tables S1–S3, supplemental notes, and supplemental material and methods

Document S2. Article plus supplemental information
