# Supplementary material for: Bi-allelic variants in FSD1L cause a neurodevelopmental disorder overlapping with L1 syndrome
Source: Am J Hum Genet. 2026 Feb 19;113(3):600–15. doi: 10.1016/j.ajhg.2026.01.014 (PMC13087397; doi:10.1016/j.ajhg.2026.01.014)
Supplement: Document S1. Figures S1–S8, Tables S1–S3, supplemental notes, and supplemental material and methods [file mmc1.pdf]

The American Journal of Human Genetics, Volume *113*

Supplemental information

Bi-allelic variants in *FSD1L* cause a neurodevelopmental disorder overlapping with L1 syndrome

Valentina Serpieri, Myriam Vezain-Mouchard, Alessia Orsi, Maryline Lecointre, Concetta Mazzotta, Florent Marguet, Anna Garbelli, Pascale Marcorelles, Ludovica Celli, Alice Goldenberg, Roberta De Mori, Nathalie Drouot, Francesco Petrizzelli, François Janin, Gaël Nicolas, Noor Smal, Claudia Condoluci, Carla Marini, Frederic Tran-Mau-Them, Valentin Ruault, Alessia Micalizzi, Silvia Bione, Tommaso Mazza, Anna Pichiecchio, Monia Ginevrino, Sarah Weckhuysen, Alice Bedois, Béatrice Desnous, Laurent Hermitte, Grace Rabie, Moien Kanaan, Bruno J. Gonzalez, Simone Sabbioneda, Annie Laquerrière, Pascale Saugier-Veber, and Enza Maria Valente

Supplemental Case Reports

Family A

Family A included two affected siblings (A.II-1 and A.II-2, male and female, now 25 and 15 years old), born from healthy Italian parents. There was no obvious consanguinity, but both parents' families originated from the same small town in central Italy.

Both siblings presented a syndromic condition characterized by psychomotor delay, severe intellectual disability with absence of acquired language, epilepsy, spastic tetraparesis, feeding and swallowing difficulties and marked reduced vision, with absent response to visual evoked potentials (cortical blindness). Cranial circumference was at the 3rd percentile in both siblings. The boy (A.II-1) also had tetralogy of Fallot, which was surgically corrected at age 6 months, while at age 21 years he received pacemaker implantation for severe bradycardia. Both siblings underwent inferior limb tenotomies during infancy and adolescence (A.II-1 at age 3, 8 and 16 years; A.II-2 at age 10 years). In addition, spasticity was treated with repeated injections of botulinum toxin, which was used also to treat sialorrhea. The brother had spine surgery to correct scoliosis ad age 14 years, and, in the immediate post-operative period, he necessitated tracheostomy and gastrostomy. He also underwent a Nissen fundoduplication at age 6 years and cholecystectomy at age 23 years. The sister had gastrostomy at age 12 years, and surgery for scoliosis at age 13 years.

Brain MRI in both siblings showed a malformative pattern characterized by hypoplastic corpus callosum, mild enlargement of lateral ventricles with irregular margins, mild reduction of posterior periventricular white matter and optic nerve hypoplasia.

Family B

Family B is a French consanguineous family. A 20-year-old woman, gravida I, para I, underwent ultrasonography (US) at 22 weeks of gestation (WG) which revealed severe

bilateral ventriculomegaly but with no other associated brain, visceral or growth parameter abnormalities (B.II-1, male fetus). Based on these findings, a medical termination of the pregnancy (TOP) was achieved at 23 WG. Chromosomal analysis performed on amniotic fluid cells revealed a normal male karyotype, 46, XY. Two additional TOPs were achieved in the following two years at 23 WG (B.II-2, female fetus) and at 22 WG (B.II-3, female fetus), for severe, apparently isolated, recurrent hydrocephalus. Since then, the mother had two other healthy children from a second union.

Growth parameters were at the 50th in the second fetus and 25th percentile in the third fetus according to the term.[4] The fetuses presented similar, although not specific, cranio-facial dysmorphisms consisting of macrocephaly, hypertelorism and broad nasal ridge, short nose with anteverted nostrils, flat philtrum, retrognathism and low set ears. Limb abnormalities consisted of camptodactyly (B.II-2) or bilateral clubfoot (B.II-2 and B.II-3), but neither adducted thumbs nor visceral and/or skeletal anomalies were observed. Brain weights were in accordance with the term despite hydrocephalus. On external examination, the Sylvian fissure largely opened. Olfactory bulbs and optic chiasm were present. On supratentorial coronal sections, ventricular dilation was severe with a considerable thinning of the cerebral mantle. The corpus callosum was absent (Figure 1Da). On sections passing through the mesencephalon, the aqueduct of Sylvius was macroscopically indiscernible (Figure 1Db). Cerebellar hypoplasia (<5th percentile) was observed in fetus B.II-3.

Histologically, the two brains displayed similar lesions. The lumen of the aqueduct of Sylvius was narrowed. Corpus callosum agenesis without Probst bundles was histologically confirmed (Figure 1De). The internal capsule was absent in B.II-2 and hypoplastic and fragmented in B.II-3, made of several small fascicles spreading into the basal ganglia (Figure 1Df). The eyes and spinal cord were structurally normal. Neither malformations nor acquired lesions were observed in any of the different infra- and supratentorial brain structures analyzed.

Family C

Family C, from Italy, included two affected male siblings born from unrelated parents. The clinical picture included severe developmental delay and intellectual disability (never acquired autonomous walking nor language), a developmental and epileptic encephalopathy characterized by infantile onset of drug-resistant epileptic spasms, tonic seizures—both spontaneous and reflex—and myoclonic seizures, progressive microcephaly, central vision deficits due to sub-regional atrophy of both optical nerves, nystagmus, strabismus, mild facial dysmorphic features (ogival palate, drooling, reduced bitemporal diameter, mild increased eyelid length, anteverted ears), sinus bradycardia, scoliosis, hypospadias, undescended testicles and precocious puberty. The two siblings died at age 14 and 16 years, due to pulmonary infective complications.

Brain imaging in both siblings showed mild ventricular dilation, cerebral atrophy mainly in the frontal regions, marked white matter reduction predominant in the posterior regions, corpus callosum and brainstem hypoplasia (images not available for publication). Magnetic resonance spectroscopy revealed decreased N-acetylaspartate levels.

Family D

This consanguineous family, coming from a small village in Morocco, included two affected individuals. The proband is a male child, born at term from healthy parents. Prenatal ultrasound disclosed agenesis of the corpus callosum. At birth, length was 48 cm (21th percentile), weight 2960 kg (17th percentile) and head circumference 32.5cm (7th percentile). He presented in neonatal life with hypotonia, global developmental delay (never acquired walking or speech), severe intellectual disability, nystagmus, spastic tetraparesis and epileptic encephalopathy. Feeding and swallowing difficulties were severe, requiring a gastrostomy. Microcephaly was progressive, with head circumference at age 7 years reaching only 46 cm (<-4.5 SD). He had some unspecific facial dysmorphisms and hypertrichosis. Brain MRI

showed agenesis of corpus callosum, marked ventricular enlargement with irregular margins mainly in the anterior portions, suspected aqueductal stenosis, white matter reduction, optic nerve and chiasm hypoplasia, cerebellar vermis and brainstem hypoplasia. He died at age 8 years from acute infectious respiratory distress. The second affected family member is a male fetus, offspring of the proband's maternal aunt. Pregnancy was terminated at 34+2 WG for cerebral malformations (corpus callosum agenesis, cerebellar hypoplasia), facial dysmorphisms (square ears, prognathism, small nose), bilateral cryptorchidism and renal hypoplasia. Parents have three other healthy children, a girl and two boys aged 12, 10 and 7, who did not undergo genetic testing.

Family E

This is a Turkish consanguineous family. The only child is a one-month-old neonate showing marked macrocephaly, signs of cranial hypertension and a severe encephalopathic picture with hypomobility. Brain MRI showed hydrocephalus with undetectable aqueduct of Sylvius and corpus callosum.

Family F

This is a Palestinian family from a consanguineous community, with first- and second-cousin marriages across generations. The couple had nine pregnancies, of which four (two males and two females) were electively terminated due to ultrasound detection of severe triventricular hydrocephalus in the fourth to fifth months of pregnancy. Genetic testing was available only for one male fetus, who underwent whole exome sequencing. A fifth pregnancy was ectopic, resulting in rupture of the fallopian tube and early miscarriage. Of the four living children, one girl has hypoparathyroidism and three are in good health. Three other affected individuals are reported in the extended family, including two female cousins and one uncle. One female cousin showed severe hydrocephalus, encephalopathy with lack of acquisition of developmental milestones and failure to thrive. She underwent a neurosurgical shunting procedure at age 1

month and died at age 1 year. The other female cousin, also presenting severe hydrocephalus and macrocephaly, died soon after birth. One uncle was also similarly affected with hydrocephalus requiring shunt surgery, and died at age two years. No additional clinical documentation or imaging is available.

Supplemental Figures

Figure S1 - Fsd1l-CRISPR/GFP plasmids used for *in utero* electroporation

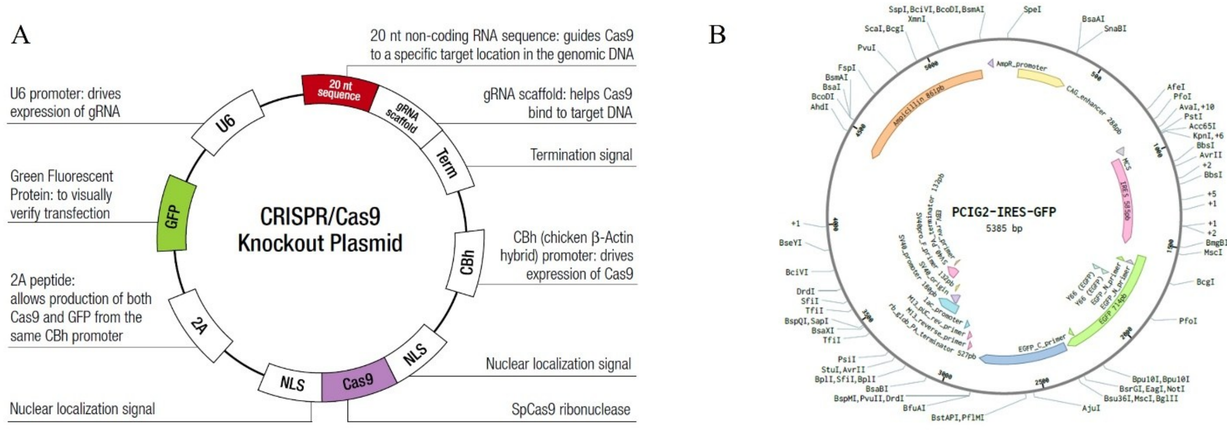

A) Map of the Fsd1l CRISPR/Cas9 knockout plasmid; B) Map of the PCIG2-IRES-GFP plasmid used to control the electroporation conditions at E15.

Figure S2 - Characterization of the impact of missense variants

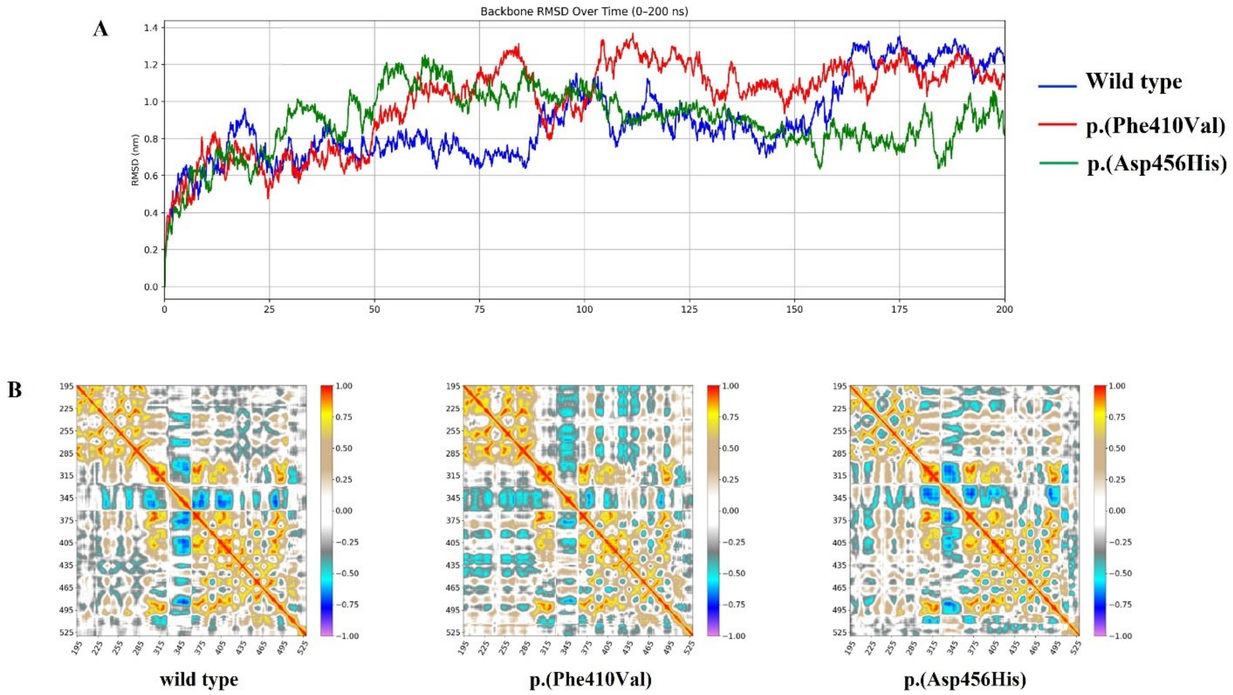

A) RMSD profiles of the heavy atoms for the wild-type (blue), p.(Phe410Val) (red) and p.(Asp456His), (green); B) DCCMs of wild-type and mutant proteins. Perfect correlations are highlighted in red (direct) or violet (inverse);

Figure S3 – Prediction of splicing defect for variant c.409T>G

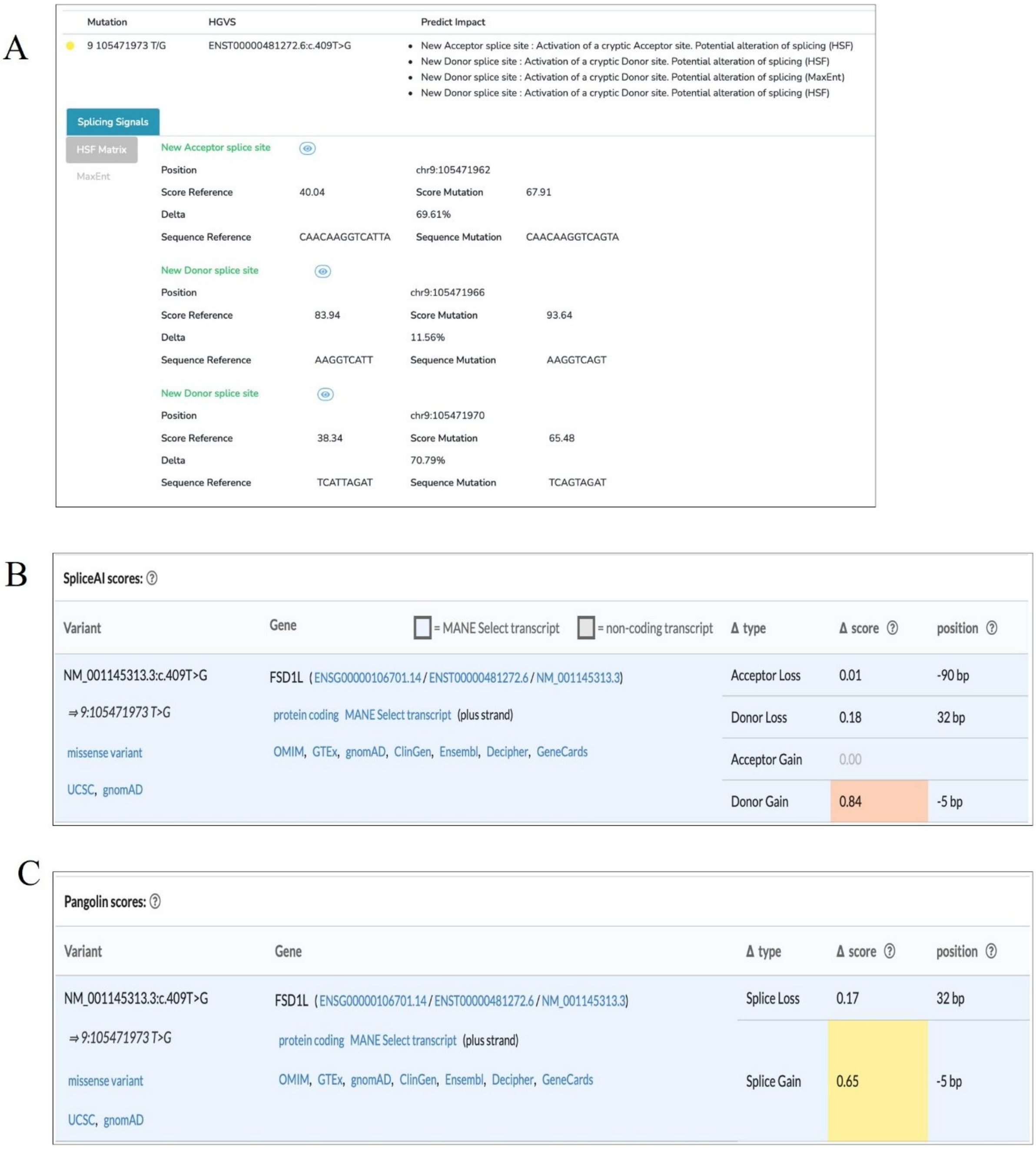

Outcome of Human Splicing Finder (A), SpliceAI (B) and Pangolin (C), consistently predicting the activation of a cryptic donor splice site.

Figure S4 – RNA studies in affected individuals from families A-B-C

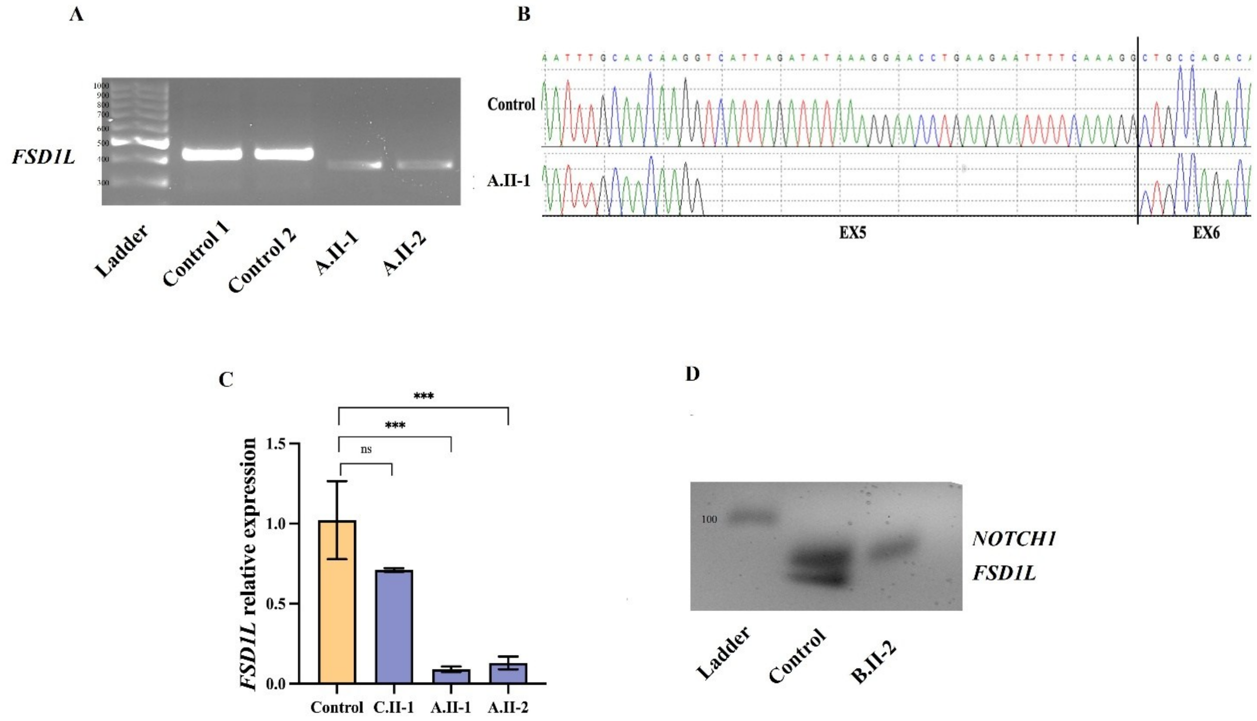

A) semiquantitative PCR amplification of a *FSD1L* cDNA fragment encompassing the c.409T>G variant from individuals A.II-1, A.II-2 and two controls; B) Electropherogram obtained upon cloning and sequencing the same fragment; C) quantitative RT-PCR showing *FSD1L* expression levels in individuals A.II-1, A.II-2 and C.II-1. p-value: ***<0.005; D) Semiquantitative PCR amplification of a short fragment of *FSD1L* and housekeeping *NOTCH1* cDNAs from fetal brain tissue of affected fetus B.II-2.

Figure S5 - Transcripts and expression pattern of *FSD1L*

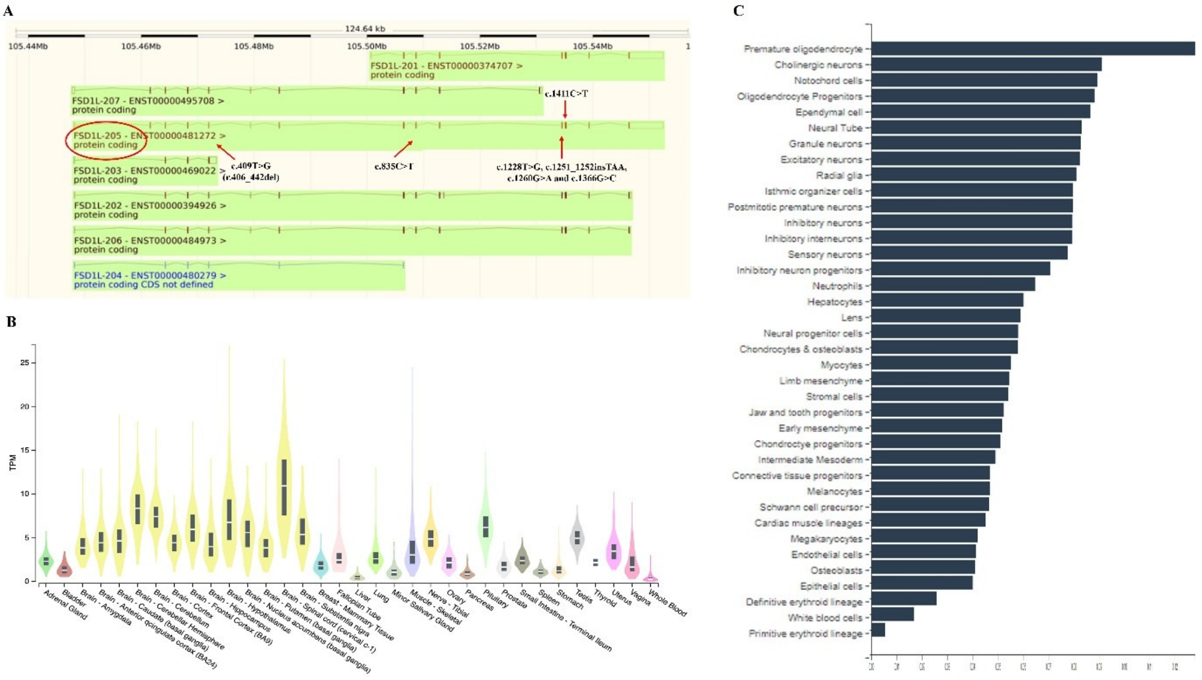

A) Transcripts of *FSD1L* according to Ensembl (205 is the canonical transcript, while 204 is non-coding) and exons affected by the identified variants; B) *FSD1L* expression from GTEx (https://www.gtexportal.org/home/) , showing highest expression in brain; C) *Fsd1l* expression in mouse embryos at 9.5-13.5 days of gestation from MOCA (https://oncoscape.v3.sttrcancer.org/atlas.gs.washington.edu.mouse.rna/landing), showing highest expression in premature and progenitor neuronal and glial populations.

Figure S6 - Immunohistochemical localization of FSD1L and L1CAM in the optic nerves and retina

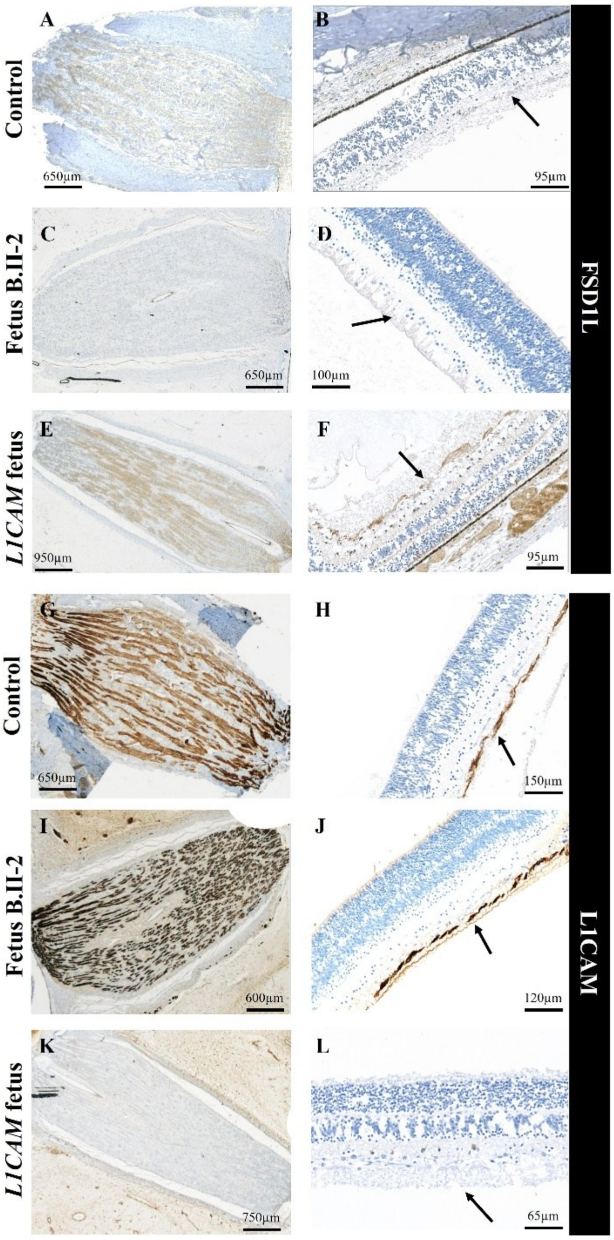

FSD1L was observed in the optic nerve (left) and axons of retinal ganglion cells (right) from control (A, arrow in B) and fetuses carrying *L1CAM* variants (E,F) while no immunoreactivity was detected in the eye of the fetus with *FSD1L* variants (C,D). A similar pattern was obtained when staining the eye of control and fetus with *FSD1L* variants using anti-L1CAM antibody (G,H and I,J), while no L1CAM was detected in the fetus mutated in *L1CAM* (K,L).

Figure S7 - *Fsd1l* repression in the developing brain in E18 mouse embryos

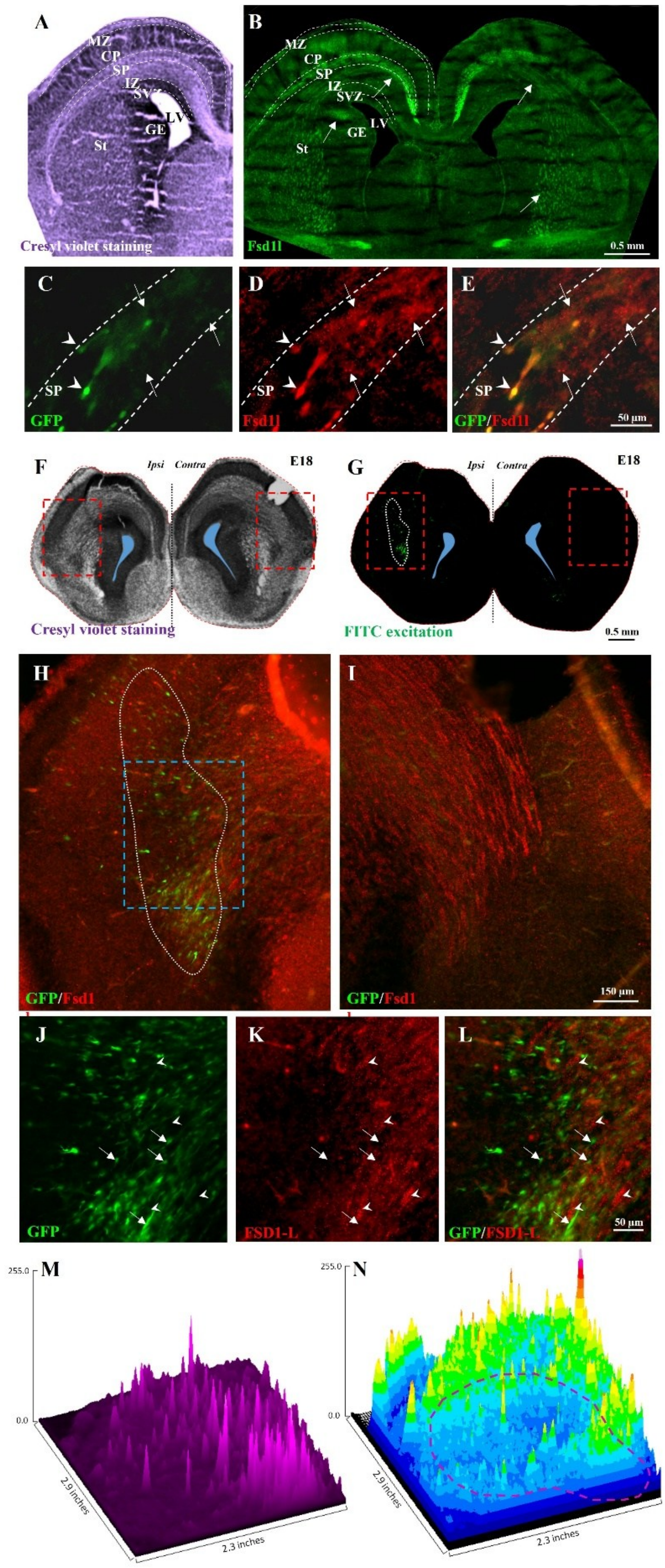

A) Cresyl violet-stained section visualizing different brain structures at E18: MZ: marginal zone; CP: cortical plate; SP: subplate; IZ: intermediate zone; SVZ: subventricular zone; St: striatum; GE: ganglionic eminence; LV: lateral ventricle. B) Fsd1l immunostaining in the developing forebrain. Note the presence of Fsd1l-positive fibers running in the SP, IZ and St (arrows). C-E) Visualization in the SP zone of a Fsd1l-positive cell (arrowhead) expressing GFP after *in utero* electroporation of a GFP control plasmid at E15. Arrows indicate Fsd1l cells/fibers negative to GFP. F-G) Low magnification microphotograph of a cresyl violet-stained section at E18 used to localize the GFP-positive area in an embryo electroporated with the Fsd1l-CRISPR/GFP plasmid at E15 (white dotted line). H-I) higher magnification of the red dotted rectangles visualizing the ipsi- and contralateral regions, with overlay of Fsd1l and GFP fluorescent signals in the ipsi- (H, electroporated) and contralateral (I) side. Note the presence of several GFP-positive cells in the electroporated sides. J-L) Higher magnification of the blue dotted rectangle. Note that GFP-positive cells are Fsd1l-negative (arrows) whereas Fsd1l-positive cells are GFP-negative (arrowheads). M,N**)** Surface plots visualizing GFP and Fsd1l intensity profiles. Low Fsd1l-intensity zone (dotted line area) coincides with high GFP-positive zone.

Figure S8 - *FSD1L* defects impair mitotic spindle formation and nuclear morphology

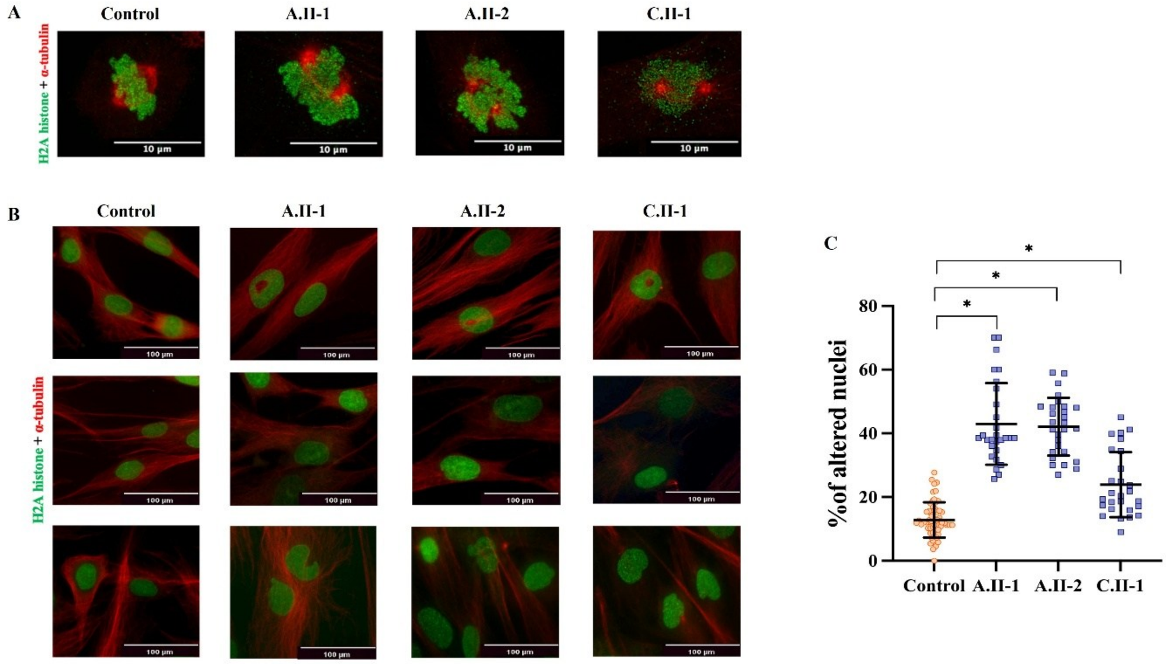

A) Additional representative images of abnormal spindle formation in fibroblasts from individuals A.II-1, A.II-2 and C.II-1 compared to control; scale bar 10μM. B) representative images of nuclear abnormalities (hollow nuclei, faded nuclei, multilobate nuclei) in fibroblasts from affected individuals; scale bar 100μM.C) graph showing the percentage of cells with nuclear abnormalities in fibroblasts of affected individuals versus controls (mean ± SE). p-value: *<0.05.

Supplemental Tables

Table S1 – *In silico* predictions of pathogenicity for the two *FSD1L* missense variants

	**p.(Phe410Val)**	**p.(Asp456His)**
SIFT	0.034 (D)	0.001 (D)
SIFT4G	0.025 (D)	0.006 (D)
Polyphen2-HDIV	0.368 (B)	0.862 (P)
Polyphen2-HVAR	0.196 (B)	0.58 (P)
MutationTaster	0.71 (D)	0.46 (N)
PROVEAN	-3.72 (D)	-5.06 (D)
MetaSVM	-0.703 (T)	0.539 (D)
MetaLR	0.251 (T)	0.669 (D)
MetaRNN	0.519 (D)	0.824 (D)
M-CAP	0.032 (D)	0.111 (D)
BayesDel-addAF	0.685 (D)	0.759 (D)
BayesDel-noAF	-0.029 (D)	0.082 (D)
ClinPred	0.988 (D)	0.98 (D)
AlphaMissense	0.990 (P)	0.719 (P)
CADD	24.8	28.3

B = Benign; D = Damaging; N = Neutral; P = Possibly Damaging; T = Tolerated

Table S2 – Gestational age and cause of death of control fetuses

Fetus number	Term	Cerebral maturation*	TOP	Cause of death
**1**	12 WG	4.9g - 12 WG	Yes	Anterior celosomia
**2**	14 WG	6g - 13 WG	Yes	Radial agenesis and oligodactyly
**3**	16 WG	16.1g -14/15 WG	Yes	Isolated sacral myelomeningocele
**4**	16 WG	17.5g - 16 WG	Yes	Mitral valve atresia
**5**	18 WG	26.6g - 17/18 WG	Yes	Isolated bilateral renal agenesis
**6**	22 WG	58.5g - 21/22 WG	No	Acute chorioamnionitis
**7**	22 WG	77g - 22/23 WG	Yes	Complex cardiac malformation

TOP: Medical termination of pregnancy; WG: weeks of gestation. *According to the morphometric criteria of Guihard-Costa and Larroche.[5]

Table S3 – Antibodies used in the study

Antibody	Dilution	Product number
Mouse TRA-1-60	1:250	#AB16288, Abcam
Rabbit OCT4	1:200	#AB19857, Abcam
Mouse SSEA4	1:250	#AB16287, Abcam
Rabbit SOX2	1:500	#AB97959, Abcam
Rabbit SOX1	1:500	#AB87775, Abcam
Mouse PAX6	1:100	#MA1-109, Invitrogen
Mouse Nestin	1:500	#AB22035, Abcam
Mouse βIII-Tubulin	1:500	#AB7751, Abcam
Rabbit HuC/D	1:250	#AB184267, Abcam
Rabbit FSD1L	1:250	#NBP1-79841, Novus Bio
Rabbit γ-Tubulin	1:500	#PA128042, Invitrogen
Mouse L1CAM	1:100 (IHC), 1:200	#AB24345, Abcam
Mouse Acetylated Tubulin	1:500	#T7451, Sigma Aldrich
Rat tyr-tub	1:500	#MAB1864-I, Sigma Aldrich
Rabbit Histon H2A	1:250	#PA5-28778, Invitrogen
Mouse α-Tubulin	1:2000	#T5168, Sigma Aldrich
Mouse ARL13B	1:250	#75-287, Antibodies Incorporated
Rabbit HA-tag	1:200	#71-5500, Thermo Fisher Scientific
Goat Anti-Mouse IgG, (DyLight 488)	1:500	#35503, Thermo Fisher Scientific
Goat Anti-Rabbit IgG, (DyLight 550)	1:500	#SA5-10033, Thermo Fisher Scientific
Goat Anti-Mouse IgG, (DyLight 550)	1:500	#SA5-10173, Thermo Fisher Scientific
Goat Anti-Rabbit IgG, (DyLight 488)	1:500	#35553, Thermo Fisher Scientific

Supplemental Materials and Methods

Gaussian accelerated Molecular Dynamics simulation

Gaussian accelerated Molecular Dynamics (GaMD) simulation method was implemented to investigate the impact of missense variants c.1228T>G and c.1366G>C on protein dynamics and conformational transitions.

Atomic coordinates of FSD1L were retrieved from the AlphaFold v.2.0 web server.[1] Only the region spanning residues 195–530 was retained, corresponding to the fibronectin type-III and B30.2/SPRY domains. Then, the wild-type structure was mutated *in silico* using ChimeraX,[2] to introduce c.1228T>G and c.1366G>C variants. Both wild-type and mutant structures were inserted into a simulation box, extending up to 15 Å, and solvated with the TIP3P water model using the CHARMM-GUI web-tool (https://www.charmm-gui.org/). Finally, an appropriate number of Na+ and Cl- counter ions were added to neutralize the overall charge of the models. In our simulation setup, the Amber ff14SB force field was employed.

Each system was first energy-minimized using the steepest descent method, followed by the conjugate gradient method. Thus, it was gradually heated and equilibrated for approximately 5 ns using a time- step of 1 fs. Electrostatic interactions were computed using the particle-mesh Ewald method, while a cutoff of 10Å was used for non-bonded short-range interactions. The temperature and pressure were set at 300 K and 101.3 kPa, respectively, using Langevin dynamics and Piston methods.

In the GaMD simulation, the boost potential has been applied in a dual-boost scheme, with two acceleration potentials applied simultaneously to the system: (i) the torsional terms only and (ii) across the entire potential. A time step of 2 fs was used. The maximum, minimum, average, and standard deviation values of the system potential were obtained from an initial ~12 ns NPT simulation with no boost potential. Each GaMD simulation proceeded with a ~50

ns run, in which the boost potential was updated every 1.6 ns, thus reaching equilibrium values. Finally, ~200 ns of GaMD simulations were carried out in triplicate.

These GaMD trajectories were analyzed from geometric and energetic points of view, excluding the preparatory steps. First, we assessed root-mean-square deviation (RMSD), a standard measure of the structural distance between coordinates that provides a quantitative measure of the structural changes that occur during the simulation. Next, we performed a Principal Component Analysis (PCA), to probe the conformational changes occurring in our systems during the simulation. We inferred large-scale collective fluctuations of atoms and predicted low-dimensional subspaces where essential protein motions were expected to occur. Thus, a covariance matrix was generated using the gmx_covar function implemented in GROMACS v2018, which captures the degree of collinearity of the atomic motions of each pair of atoms. The conformational changes caused by the variants under investigation were explored using Dynamic Cross-Correlation Maps (DCCMs). These were plotted using a custom Python script that takes covariance matrices as inputs and generates correlation matrices. DCCMs allowed us to study the long-range interactions between all pairs of atoms and highlight any correlated and anticorrelated motion. Finally, the GetContacts tool (https://github.com/getcontacts/getcontacts) was utilized to rapidly compute and compare the frequency of interaction during each trajectory.

Characterization of impact of variant c.409T>G on splicing

Total RNA from fibroblasts of individuals A.II-1 and A.II-2 (family A) and 3 healthy controls was retrotranscribed. A primer pair was designed to amplify a 400bp-region containing variant c.409T>G using FIREPol® Taq (Carlo Erba, Cornaredo, Italy). The resulting amplicon was ligated into a pGEM®-T easy vector (Promega Corporation, Madison, WI, USA). Heat shock transformation of the obtained plasmid was performed in One Shot TM TOP10 Chemically Competent E. coli bacteria (Thermo Fisher Scientific, Waltham, MA,

USA). Colony PCR was performed using SP6 and T7 primers flanking the cloning site, and the obtained fragments were Sanger sequenced.

Immunoreactivity of FSD1L and L1CAM in the brain and eye

Seven control fetuses were selected to study the physiological localization of FSD1L and L1CAM during development (Supplementary Table S2). Gestational age was estimated according to biometric data, skeletal measurements and histological maturation of the brain and viscera. For immunohistochemical (IHC) studies, paraffin-embedded brain and eye sections were cut at 6-µm. Induced epitope retrieval included a microwave pre-treatment protocol (pretreatment CC1 kit, Ventana Medical Systems Inc, Tucson AZ, USA).
Incubations with the primary antibodies FSD1L and L1CAM (Supplementary Table S3) were carried out for 32 minutes at room temperature using the Benchmark XT system (Ventana Medical Systems). After incubation, slides were processed using the Ultraview Universal DAB detection kit (Ventana Medical Systems) and counterstained with hæmatoxylin. Negative controls were obtained by omission of the primary antibody or using other antibodies of known reactivity.

Immunohistochemical localization of FSD1L and L1CAM was also studied in fetuses B.II-2 and B.II-3 harboring truncating variant c.1411C>T (family B), and in two male fetuses interrupted at 22 WG, hemizygous for *L1CAM* truncating variants.

Fsd1l-CRISPR/Cas9 *in utero* electroporation

Mice were ordered from the National Marine Research Institute (Janvier, Le Genest-Saint-Isle, France) and used according to the French Ethical Committee recommendations and European directives 2010/63/UE. Pregnant mice at GD15 (E15 embryos) were anaesthetized by means of Vetflurane® inhalation for a maximum of 40 min (MiniHUB V2.1, TemSega, Pessac, France). After laparotomy, the exposed uterine horn was kept moist with a warmed physiological solution. During surgery, the body temperature of the mouse was controlled and

maintained using a hotplate (Homeothermic Monitoring System, Harvard Apparatus, Holliston, MA, USA). Unilateral intraventricular injections were performed using glass capillaries (0.58 mm inner diameter, 1.0 mm outer diameter, Harvard apparatus) with a P-97 flaming/brown micropipette puller (Sutter Instrument Company, Novato, CA, USA).

The Fsd1l-CRISPR/GFP electroporated group received injection of both the Fsd1l-CRISPR/Cas9 KO plasmid (0.5 µg/µL; sc-435647, Santa Cruz Biotechnologies, Dallas, TX, USA) and the PCIG2-IRES-GFP plasmid (0.5 µg/µL; generous gift from Polleux F)[3], while the GFP electroporated group received the PCIG2-IRES-GFP plasmid alone (Supplementary Figure S2). The injection depth within the ventricle was 0.5 mm, and 1 µL of the solution with Fast Green (0.05 % PBS 1X; Sigma, Saint Louis, MO, USA) was injected. For electroporation, the appropriate voltage was applied *via* specialized platinum electrodes Nepagene CUY 650P3 (Nepagene Co., Ichikawa, Japan) with the following parameters: interval cycle length 50 msec, interval pause 950 msec. The voltage conditions were controlled on the NEPA21 type II Electroporator (Nepagene Co., Ichikawa, Japan). After electroporation, the abdominal walls were sutured with sterile Silk Suture Prolene 6-0, MPP2832 (ETHICON, Lindingö, Sweden).

Three days after *in utero* electroporation (E18), the brains were collected for anatomical, immunohistochemical and image analyses. The brains were immersed in 0.1 M phosphate-buffered saline (PBS) containing 4% paraformaldehyde (PFA) for 24 h at 4°C, then incubated overnight in 30% sucrose and frozen in isopentane (-40°C). Coronal sections (25 µm thick) were cut on a cryomicrotome (Leica Microsystems, Nanterre, France) and stored at -80°C until use. Slices were incubated overnight at 4°C with FSD1L primary antibody (Supplementary Table S3) diluted in an incubation buffer (PBS containing 1% BSA and 3% Triton X-100). Next, slices were rinsed twice with PBS for 20 min and incubated in the same buffer containing the appropriate secondary antibody for 2 hours at room temperature.

Fluorescent signals were observed with a Leica DMI 6000B microscope (Leica Microsystems) and images saved as Tiff format. The specificity of the immunoreaction was controlled by omitting the primary antibody. Intensity profile areas were obtained using the *Surface plot* tool of the ImageJ software (National Institutes of Health, Bethesda, MD, USA). Twenty-five micrometer serial transverse sections were performed in the forebrain of control, PCIG2-IRES-GFP and Fsd1l-CRISPR/GFP embryos. After Cresyl violet staining, areas of the ipsi- and contro-lateral ventricles were measured using the Metamorph® software (Roper Scientific, Tucson, AZ, USA). Data obtained from each section were then integrated giving access to the lateral ventricle volume.

Statistical analyses were performed using the biostatistics Prism software (GraphPad Inc., La Jolla, CA, USA). Frequencies of mice with dilated lateral ventricles (dLV) in the control, PCIG2-IRES-GFP and Fsd1l-CRISPR/GFP groups were analyzed using the Chi-square test. Comparison of the ipsi- and contralateral sides of the dLV was done using the unpaired t-test.

Generation of the FSD1L-HA *knock-in* iPSC line

Briefly, 2.0×10^5 mycoplasma-free iPSCs (HDF109) were nucleofected, with Alt-R S.p. Cas9 Nuclease V3, one sgRNA at 3' of *FSD1L* sequence (upstream of the stop codon), Alt-R Cas9 Electroporation Enhancer and Ultramer DNA oligonucleotide, all purchased by Integrated DNA Technologies (IDT, Newark, NJ, USA). Next, iPSCs were seeded in a vitronectin-coated 4 well-plate for a week, after that cells were diluted and plated into 6-well plates. Formed colonies were picked and grown as single colonies. PCR reactions on genomic DNA extracted from iPSCs were used to rapidly confirm the correct insertion of HA-tag in either homozygous or heterozygous state. Primer sequences are available upon request.

Supplemental References

1. Jumper, J., Evans, R., Pritzel, A., Green, T., Figurnov, M., Ronneberger, O., Tunyasuvunakool, K., Bates, R., Žídek, A., Potapenko, A., et al. (2021). Highly accurate protein structure prediction with AlphaFold. Nature *596,* 583-589. https://doi.org10.1038/s41586-021-03819-2.
2. Pettersen, E.F.G., T.D.; Huang, C.C.; Meng, E.C.; Couch, G.S.; Croll, T.I.; Morris, J.H., Ferrin, T.E. (2021). UCSF ChimeraX: Structure visualization for researchers, educators, and developers. Protein Sci *30,* 70-82. https://doi.org10.1002/pro.3943.
3. Hand, R., Bortone, D., Mattar, P., Nguyen, L., Heng, J.I., Guerrier, S., Boutt, E., Peters, E., Barnes, A.P., Parras, C., et al. (2005). Phosphorylation of Neurogenin2 specifies the migration properties and the dendritic morphology of pyramidal neurons in the neocortex. Neuron *48,* 45-62. https://doi.org10.1016/j.neuron.2005.08.032.
4. Guihard-Costa, A.M., Menez, F., Delezoide, A.L. (2002). Organ weights in human fetuses after formalin fixation: standards by gestational age and body weight. Pediatr Dev Pathol *5,* 559-578. https://doi.org10.1007/s10024-002-0036-7.
5. Guihard-Costa, A.M., Larroche, J.C. (1990). Differential growth between the fetal brain and its infratentorial part. Early Hum Dev *23,* 27-40. https://doi.org10.1016/0378-3782(90)90126-4.
